# Interdomain dynamics in human Replication Protein A regulates kinetics and thermodynamics of its binding to ssDNA

**DOI:** 10.1371/journal.pone.0278396

**Published:** 2023-01-19

**Authors:** Sangeeta, Arnab Bhattacherjee

**Affiliations:** School of Computational and Integrative Sciences, Jawaharlal Nehru University, New Delhi, India; Weizmann Institute of Science, ISRAEL

## Abstract

Human Replication Protein A (hRPA) is a multidomain protein that interacts with ssDNA intermediates to provide the latter much-needed stability during DNA metabolism and maintain genomic integrity. Although the ssDNA organization with hRPA was studied recently through experimental means, characterizing the underlying mechanism at the atomic level remains challenging because of the dynamic domain architecture of hRPA and poorly understood heterogeneity of ssDNA-protein interactions. Here, we used a computational framework, precisely tailored to capture protein-ssDNA interactions, and investigated the binding of hRPA with a 60 nt ssDNA. Two distinct binding mechanisms are realized based on the hRPA domain flexibility. For a rigid domain architecture of hRPA, ssDNA binds sequentially with hRPA domains, resulting in slow association kinetics. The binding pathway involves the formation of stable and distinct intermediate states. On contrary, for a flexible domain architecture of hRPA, ssDNA binds synergistically to the A and B domains followed by the rest of hRPA. The domain dynamics in hRPA alleviates the free energy cost of domain orientation necessary for specific binding with ssDNA, leading to fast association kinetics along a downhill binding free energy landscape. An ensemble of free energetically degenerate intermediate states is encountered that makes it arduous to characterize them structurally. An excellent match between our results with the available experimental observations provides new insights into the rich dynamics of hRPA binding to ssDNA and in general paves the way to investigate intricate details of ssDNA-protein interactions, crucial for cellular functioning.

## Introduction

The Replication Protein A (RPA) is a eukaryotic heterotrimeric protein that avidly binds with ssDNA to orchestrate crucial DNA metabolic processes. Since its first characterization as an essential factor for the in vitro replication of Simian virus 40 (SV40) DNA [1–8], human RPA (hRPA) has emerged as the key target and an important regulator of the genome maintenance machinery [9–11]. Following cellular exposure to genotoxic stresses, hRPA also acts as a key sensor to elicit the DNA damage response (DDR) [9, 12–16], triggers DNA recombination, base excision repair, and the nucleotide excision repair process [17–20] by binding with heterogeneous protein partners such as RAD52, Uracil DNA glycosylase 2, and XPA respectively. The common trait in all these DNA metabolic processes is the involvement of hRPA and other proteins [21] with ssDNA intermediate that prevents the formation of unwanted secondary structure (e.g., G-quadruplex [22, 23] in ssDNA, thereby shielding the DNA strands from endonuclease activity and preserving genomic integrity. Conjectures have been made about how hRPA manages to regulate such a diverse array of functions [18], but uncertainty prevails as there is no structural framework to delineate the functions of the whole hRPA complex while interacting with ssDNA.

**Fig 1:**
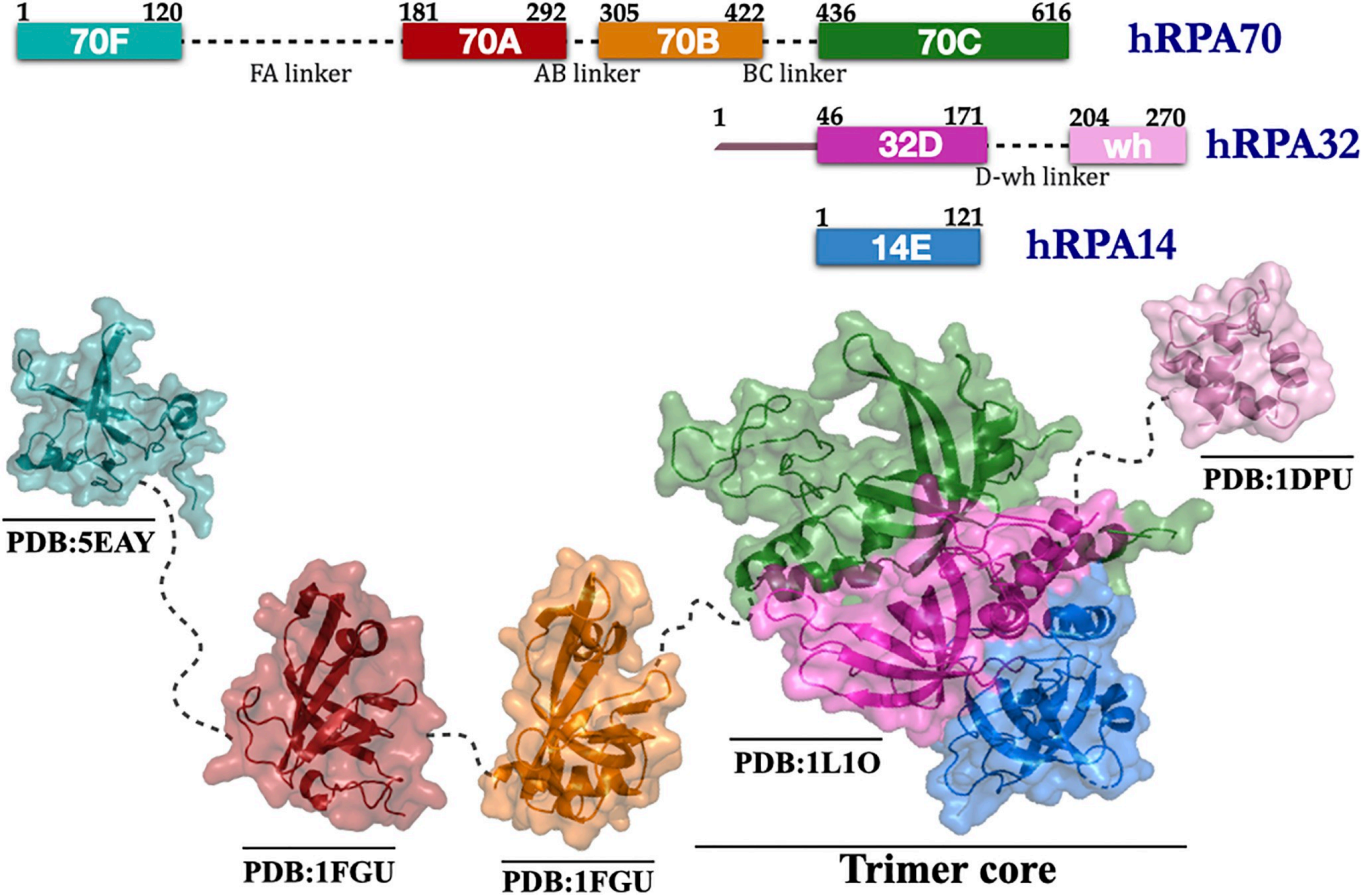
Structural characterization of multidomain human Replication Protein A. Top panel: The domain composition and organization of hRPA’s three subunits (hRPA70, hRPA32, hRPA14) along with their residue number and respective linkers. The largest subunit hRPA70 is composed of DBD-F, DBD-A, DBD-B, and DBD-C. hRPA32 subunit consists of DBD-D along with a disordered N-terminal region (32N) and a C-terminal domain that adopts a winged-helix-turn-helix fold (WH). hRPA14 subunit consists of DBD-E. hRPA70C, hRPA32D and hRPA14E domain together forms the trimer core of hRPA. The remaining domains in the subunits are connected by flexible linkers. Bottom panel: The crystal structures for hRPA70F, hRPA70AB, hRPA70C/32D/14E, and hRPA32C modules is depicted here along with their PDB IDs as obtained from the RCSB Protein Data Bank. The domains and linkers are colored to match the domains as shown in top panel.

The crux of the problem is the difficulty associated with the characterization of heterogeneous ssDNA-protein interactions and the lack of understanding of the modular nature of hRPA architecture. While the first issue has been investigated previously [24–26] and it was shown that aromatic and hydrogen bond interactions also play significant roles besides electrostatic interactions to achieve the binding specificity, the latter issue remains unexplored. hRPA comprises three subunits named hRPA70, hRPA32, and hRPA14 of varying sizes and molecular weights. The detailed structure further reveals that the three subunits are composed of six distinctly folded domains, which adopt the oligonucleotide/oligosaccharide binding fold [13, 27, 28] (OB-fold; denoted from A to F), a signature architectural trait of many other ssDNA-binding proteins (SSBs). The largest subunit hRPA70 is composed of DNA binding domains (DBDs), DBD-F, DBD-A, DBD-B, and DBD-C. hRPA32 subunit consists of DBD-D along with a disordered N-terminal region (32N), which is the primary phosphorylation site of the protein, and a C-terminal domain that adopts a winged-helix-turn-helix fold (WH) (RPA32C) [17]. The smallest subunit hRPA14 spans over the entire DBD-E region. Among all the domains, DBD-F and WH are primarily involved in protein interactions [9, 10, 18, 29, 30] whereas DBD-A, B, C & D actively participate in interacting with ssDNA [18]. The schematic presented in Fig 1 represents the complex domain organization of hRPA. The overall structure of hRPA can be organized into five distinct structural modules, 70F, 70AB, 70C/32D/14E, 32N, and WH. Except 32N, the structure of the above-mentioned modules have been determined individually at atomic resolution and their respective biochemical contribution to protein-protein interaction and ssDNA binding have been characterized [17, 31–38]. The structure of the full hRPA is, however, yet to be solved. The individual protein domains are interconnected via long, flexible linker regions that indicate the possibility of large range domain movement and extensive domain-domain crosstalks. Indeed, previous analysis of full-length hRPA, DNA binding core of hRPA, and tandem domain fragments by NMR and SAXS study have revealed that DBDs of hRPA adopt a myriad inter-domain orientation and result in significant diversity in its interactions with ssDNA and other protein partners [39–42]. For instance, individually DBD-A and DBD-B are noted to exhibit high-affinity towards ssDNA with equilibrium dissociation rate constant (*k*_*d*_) values of 2 *μ*M, and 20 *μ*M [34, 39, 43]. However, together these two domains feature a much lower *k*_*d*_ value of 50 *n*M [34, 39, 43], indicating that the linkage between two weak DBDs may promote inter-domain interactions and thereby enhance the overall affinity of hRPA by several orders of magnitude. Another manifestation of the heterogeneous inter-domain interactions is the two different modes of association observed for hRPA-ssDNA based on the length of the latter and the number of participating DBDs of hRPA [18, 33, 36, 41, 44–50]. One mode involves the high-affinity domain pair 70AB engaging 8–10 nt of ssDNA, while in another mode, low-affinity domains 70C and 32D also get involved along with 70AB, extending the occluded site size to 24–30 nt [31–34, 39, 43–45, 51, 52].

Notwithstanding the wealth of information available regarding the structure and function of these domains at an individual level, the molecular principle of hRPA binding to ssDNA and the underlying mechanism of a dynamic association of its domains remain elusive. In particular, how the inter-domain dynamics of hRPA influence the coordination between the protein and ssDNA intermediates, is still unknown. To address this gap, we probed the hRPA binding to a 60 nt ssDNA using a coarse-grained (CG) protein—ssDNA model, where the native fold of the protein was maintained through a Gō-like potential and the ssDNA was designed following the 3SPN.2 DNA model, originally developed by Hinckley *et al*. [53]. The computational framework is developed to unravel the impacts of conformational heterogeneity of both the molecules on their binding mechanism. We monitored the domain-domain movement by modulating the strength of the inter-domain interactions. The resultant conformational heterogeneity is compared with the ensemble-averaged structural parameters obtained from SAXS and all-atom simulation data to mimic the inter-domain flexibility of the entire hRPA molecule. Our results suggest that the inter-domain dynamics of the hRPA molecule plays a crucial role in modulating the interactions with ssDNA, favouring the anchoring of the latter to hRPA. The corresponding kinetic study confirms a faster association rate between ssDNA and hRPA when the individual domains of the latter are flexible to adapt to conformational changes induced by the progression of ssDNA binding. Furthermore, our result suggests the binding proceeds through several degenerate intermediate states, depicting a nearly downhill free energy landscape of association. The degeneracy of the intermediate states make it difficult to characterize them precisely. The result is consistent with the consensus view that hRPA engages ssDNA in initial, intermediate, and final stages although the intermediate binding mode remained poorly characterized [18, 41]. Our results provide new insights on how the coupling of hRPA domain dynamics and DNA-binding activities drive the changes in the overall architecture of the protein complex and regulate the progression of DNA processing machinery.

## Materials and methods

### Protein model

In this study, the coarse-grained (CG) descriptions for protein and ssDNA were used. hRPA was selected as a model system for the protein that preferentially binds with a polypyrimidine ssDNA sequence. The structure of the full hRPA is, however, yet to be solved. Therefore, we modeled the full hRPA structure using its individually resolved modules and placed them on the template of a ssDNA bound *U. maydis* RPA crystal structure [37] (PDB ID: 4GNX & 4GOP). The crystal structures for hRPA70F, hRPA70AB, hRPA70C/32D/14E, and hRPA32C were obtained from the RCSB Protein Data Bank [PDB ID: 5EAY [54], 1FGU [33], 1L1O [34], 1DPU [17] respectively]. Modeller v9.17 (https://salilab.org/modeller/) [55] was used to model the missing loops and linkers between hRPA70F-hRPA70A, hRPA70B-hRPA70C, hRPA32D-hRPA32C domains of hRPA. Each amino acid in the protein was represented by a single bead of radius 2 Å placed at their respective *C*_*α*_ position [56–58]. The energetics of the protein molecule was described by a native topology-based model [59, 60] that uses the Lennard-Jones potential to represent native contact interactions. Such structure-based potential has been extensively used to understand the biophysics of protein-protein [61] and protein-DNA interactions [26, 62–68]. The electrostatic interactions between the charged residues were modeled using Debye-Hückel Potential. The details of the explicit form of force field and the parameters used for protein modeling (see S1 Table in Supporting Information).

### ssDNA model

The geometry of a 60 bp B-DNA was generated using w3DNA 2.0 webserver (3D DNA Structure webserver) [69] with poly T sequence. In our study, we used only a single strand of this DNA (poly T ssDNA) and its complementary strand was discarded. We adopted 3SPN.2 coarse-grained model of DNA developed in de Pablo’s group [53]. Here, the all-atom structure of ssDNA was reduced to a coarse-grained representation using the resolution of three beads per nucleotide that are placed at the geometric centres of sugar, phosphate, and nucleobase respectively. This model has achieved success in predicting the structural and mechanical properties of ssDNA including the persistence length and the sequence dependent flexibility of ssDNA in accordance with the experimental measurements. The details of the energy functions, and the parameters used for ssDNA modeling (see S2 Table) are obtained from 3SPN.2 model for DNA [53], and are described in detail in supporting information.

### Sequence-dependent model for protein-ssDNA interactions

In our model, we incorporated the following four interaction potentials between the protein and ssDNA molecule to study their binding at the molecular level: (i) electrostatic interaction between the charged amino acids of protein and negatively charged phosphate beads of ssDNA. The ssDNA phosphate beads were assigned with a net charge of -0.6 to consider the effects of counterion condensation. The electrostatic interactions were modeled by the Debye-Hückel potential. Despite the limited applicability of Debye-Hückel potential at low ionic concentrations, the applications of this potential have been widely studied in the various nucleic acid and protein biophysics [70, 71]. The effective strength of protein and DNA interaction is scaled by a factor of 1.67 to bring the local charge of phosphate beads back to -1 as used by Lequieu *et al*. [72]. Furthermore, it should be noted that the strength of the electrostatic interactions in the CG description of a system is generally lower compared to that in atomistic models under identical salt conditions. This is due to longer distance between charge carrying beads in the CG framework compared to that in the atomistic description, where amino acid side chains and their rotational degrees of freedom (rotamers) allow a much closer approach of the charge bearing atoms. Since protein-ssDNA interactions are primarily driven by electrostatic interactions, a low salt condition is necessary to bring them in close contact and study their interaction dynamics in a CG description. We note that similar salt condition was used successfully in previous studies to investigate protein-ssDNA interactions [24, 25, 73] with a coarse-grained model. (ii) the aromatic stacking interaction between the aromatic amino acids of protein (Phe, His, Tyr, Trp) and ssDNA bases, (iii) the hydrogen bond interactions between non-aromatic amino acids (hydrophobic and polar both) and the nucleobases of ssDNA, and (iv) the excluded volume interaction between the protein and ssDNA beads. To consider this excluded volume effect between the beads, a purely repulsive interaction was applied. The aromatic stacking, hydrogen bond and excluded volume interactions were modeled by the Lennard-Jones potential with different interaction strengths. The details of the explicit form of force field [26] and the parameters used to model protein ssDNA interactions (see S3 Table in Supporting Information). To this end, it is important to note that the potential energy functions of our coarse-grained model for protein–ssDNA binding are fully transferable and free from any bias toward the final complex formation. Indeed, the developed force-field has been extremely successful in describing binding of multiple protein-ssDNA interactions in our previous study irrespective of ssDNA binding motifs [26]. The model has previously been used to successfully capture the bound state conformations of a number of protein-ssDNA complexes irrespective of the length and sequences of associated ssDNA molecule. A list of the same and the Root Mean Squared Deviations (RMSD) between their crystal structures and our simulation predicted conformations starting from a completely unbound conformations are given in (see S4 Table) in Supporting Information. Furthermore, a detailed list of sources for various model parameters is given in S5 Table in Supporting Information.

### Simulation protocol

In the present study, the coarse-grained nature of the model was adopted to investigate apo hRPA and hRPA-ssDNA dynamics. Intra-domain and inter-domain contacts in modeled hRPA were identified by applying a distance criterion of 6.5 Å between the non-hydrogenous atoms of *i*^*th*^ and *j*^*th*^ amino acids of the all-atom protein structure within the same and different domains respectively. The inter-domain strength (*ϵ*_*inter*_) was modulated from 1.0 to 0.3 kcal/mol keeping intra-domain strength (*ϵ*_*intra*_) fixed at 1.0 kcal/mol. The simulation was performed by initially placing apo hRPA in the middle of a simulation box of dimension 230 × 245 × 250 Å with the periodic boundary condition. The unbiased binding simulations of the hRPA-ssDNA system were performed by initially placing the 60 nt ssDNA at a distance of 50 Å from the hRPA surface in the simulation box of dimension 320 × 300 × 300 Å with the periodic boundary conditions. The time evolution of their motion was studied through Langevin dynamics, where the friction coefficient, *γ*, was set to 0.1 kg/s at a temperature of 291 K and a salt concentration of 10 *m*M. We implemented the Langevin thermostat to maintain the temperature and the system was simulated using a dielectric constant of water (value = 78). The simulations were carried out to sample an NVT ensemble using an implicit solvent approach. Further, to maintain the thermal equilibrium, we discarded the initial 10^4^ steps from the trajectory. All our production runs were 2 × 10^8^ Molecular Dynamic (MD) time steps long, during which the dynamics of apo hRPA and hRPA-ssDNA are monitored by varying the inter-domain interaction strengths similar for both hRPA systems. The sufficient long timescale allows us to study the sampling of the conformational spaces. We performed 20 independent runs to investigate the detailed mechanism with acceptable statistical significance using our in-house code on a 7.74 teraflop High Performance Cluster.

### Normal Mode Analysis

To probe the large-scale functional motions of the modeled hRPA and its dynamics and flexibility, we performed the Normal Mode Analysis (NMA). For NMA analysis, the initial structure of the protein was selected after discarding the initial few steps at *ϵ*_*inter*_ value of 0.3 kcal/mol and 1.0 kcal/mol. Here, we used the coarse-grained structure of ssDNA binding core domains only (domains A-E). To perform the NMA analysis and produce the visualizations of the data, the Bio3D package in R was used [74]. A variety of functions from the Bio3D package were employed. Some of them are *nma.pdbs()*, *dccm.nma()*, and *pymol()*. The function *nma.pdbs()* was used to calculate the normal modes and produce an NMA object for visualization. *dccm.nma()* function was used to calculate the cross-correlation between DBDs. This function utilizes the normal mode analysis of a protein structure to calculate a cross-correlation matrix with elements *C*_*ij*_. Graphically, it is represented by dynamic cross-correlation map (DCCM). The *C*_*ij*_ value corresponds to the fluctuations of residues *i* and *j* and denotes if they are correlated, anti-correlated or uncorrelated. For example, *C*_*ij*_ = 1 signifies their fluctuations are completely correlated, whereas, a completely anti-correlated fluctuations of two interacting beads is represented by *C*_*ij*_ = -1. *C*_*ij*_ = 0 indicates that the fluctuations of two beads are not correlated. The Bio3D package also allows the visualization of the normal modes either through a vector field representation or trajectory file in PyMol using the Bio3D inbuilt function, *pymol()*. With this function, three-dimensional visualization of the protein is obtained and it highlights the domain dynamics in the hRPA molecule. The total number of modes generated were 2055 including the first 6 modes with zero frequency (trivial modes). These trivial modes correspond to the rotational and translational modes. For comparison between the normal modes of the protein in apo hRPA and bound hRPA conformations obtained from MD simulation trajectory (where binding is complete), we calculated the root-mean-square inner product (RMSIP) of the normal modes obtained from normal mode analysis of both conformations. These calculations were performed to facilitate a direct comparison of the flexibility patterns between protein structures. To determine which normal modes contribute to the observed conformational changes in hRPA protein from our MD simulation trajectory, the squared overlap analysis was carried out. Here, the squared overlap of the first 20 modes was calculated, with the conformational difference vector between the first (after discarding the initial 10^4^ steps) and last frames (the bound hRPA-ssDNA conformation) of our MD trajectories. The trajectory of MD simulation consists of the unbiased binding simulation of hRPA and ssDNA to achieve the final bound complex of ssDNA and hRPA (bound state) starting from both molecules in solution (unbound state). All these calculations were performed using the Bio3D package in R [74–76].

## Results

In order to untangle the role of inter-domain dynamics of hRPA in its ssDNA binding activity that trigger several critical DNA metabolic events, we first tuned the strengths of intra and inter-domain contacts present in hRPA. The hRPA molecule was modeled using its individually resolved domains that were placed together on the template of a DNA bound crystal structure of *U. maydis* RPA [37]. The strength of intra-domain contacts *ϵ*_*intra*_ was set to 1.0 kcal/mol to ensure consistency between simulated and experimentally resolved structures of the individual domains as evident from the associated RMSD plot of the hRPA domains in S1 Fig. The strength of the inter-domain contacts *ϵ*_*inter*_ was systematically varied from 1.0–0.3 kcal/mol. For each *ϵ*_*inter*_ value, we characterized the architecture of ensemble averaged conformations of hRPA and compared the same with that obtained from a SAXS experiment [41] and all-atom molecular simulation study [41]. A suitable *ϵ*_*inter*_ that could correctly represent the conformational heterogeneity of hRPA domains was recognized and adapted to further investigate how the hRPA domain dynamics influence the thermodynamics and kinetics of association with ssDNA in the rest of the study. We also used the *ϵ*_*inter*_ value of 1.0 kcal/mol as a controlled simulation that produces highly restricted domain dynamics in hRPA. Probing the hRPA-ssDNA interactions under this condition and comparing the same involving a hRPA model that captures the inter-domain flexibility of apo hRPA state allows us to directly evaluate the role of domain-domain crosstalk/interactions and flexibility in regulating the DNA processing machinery. A brief description regarding our hRPA protein model validation is given in the supporting information.

### hRPA exhibits high conformational diversity in its apo state

**Fig 2:**
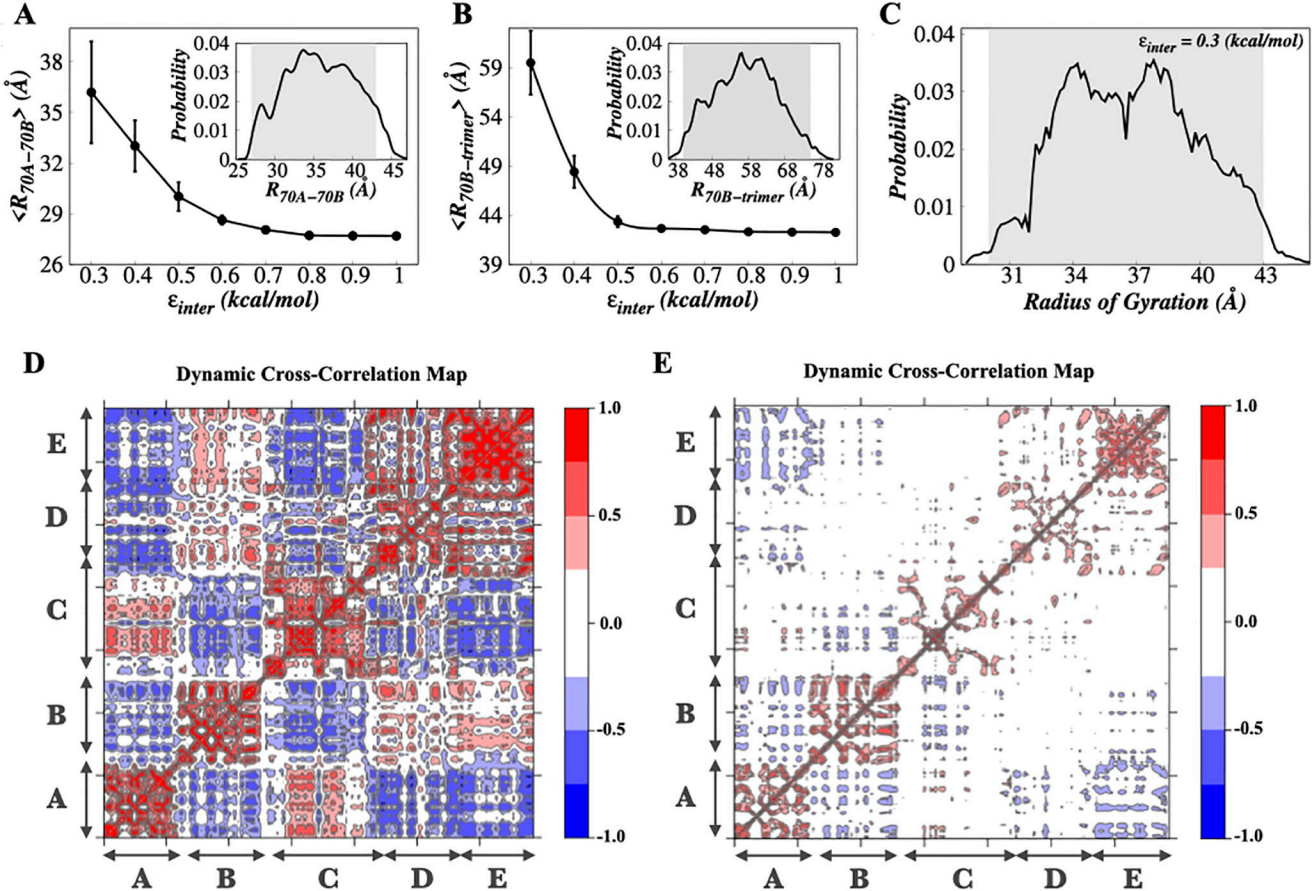
Domain-domain movement and conformational heterogeneity of hRPA. (A) Variation in the average value of inter-domain distances between DBD-A and DBD-B (< *R*_70*A*−70*B*_ >) (Å) as a function of *ϵ*_*inter*_ presented with the error bars. (B) Variation in the average value of inter-domain distances between DBD-B and the trimer core (< *R*_70*B*−*trimer*_ >) (Å) as a function of *ϵ*_*inter*_ presented with the error bars. Error bars represent standard errors; when not visible, they are smaller than the point size used here. The insets in (A) and (B) represents the probability distribution of inter-domain distances between DBD-A and DBD-B (*R*_70*A*−70*B*_) and inter-domain distances between DBD-B and the trimer core (*R*_70*B*−*trimer*_) at *ϵ*_*inter*_ = 0.3 kcal/mol. The shaded regions represent the reported range of these interdomain distances obtained from an atomistic simulation. In (A) and (B), we have presented the average and the standard errors of the inter-domain distances as a function of *ϵ*_*inter*_, whereas in the inset the spread of the same at *ϵ*_*inter*_ = 0.3 kcal/mol is presented. (C) The probability distribution of radius of gyration (*R*_*g*_) at *ϵ*_*inter*_ = 0.3 kcal/mol. The shaded region represents the reported range of ensemble averaged radius of gyration obtained from an atomistic simulation. (D, E) The dynamic cross correlation maps (DCCM) averaged over all modes for the correlation between inter-domain fluctuations for the ssDNA binding core domains of apo hRPA at the interdomain interaction strength of (D) 0.3 kcal/mol and (E) 1.0 kcal/mol. The ssDNA binding domain regions are labeled and shown by the two headed arrow.

We considered a full-length hRPA which includes DBD-F and WH along with the main DNA binding domains (DBDs A-E) (Fig 1). The N-terminal domain of hRPA32 was not taken into consideration as the segment is primarily known for phosphorylation in hRPA and is involved in the interaction with other proteins and not with ssDNA substrate [29]. The WH and DBD-F domains are connected by long flexible linkers. The DBDs (DBD-A, DBD-B and DBD-C) are tethered with each other via flexible linkers of varying lengths. Previous analysis of full-length hRPA, DNA binding core of hRPA, and tandem domain fragments by NMR and SAXS revealed that hRPA samples a wide range of diverse conformations in the absence of ssDNA. Among the hRPA domains DBD-F, DBD-A, DBD-B are considered conformationally more flexible compared to DBD-C, DBD-D, and DBD-E that together constitute the trimer core [77]. To capture the inter-domain flexibility in hRPA, we varied *ϵ*_*inter*_ from 1.0 kcal/mol to 0.3 kcal/mol at the interval of 0.1 kcal/mol keeping *ϵ*_*intra*_ fixed at 1.0 kcal/mol. To test if our model effectively captures the structural variability of hRPA due to its inter-domain flexibility and dynamics, we estimated the ensemble averaged distances between DBDs A and B (< *R*_70*A*−70*B*_ >) and DBD-B and trimer core of hRPA (< *R*_70*B*−*trimer*_ >). In Fig 2A and 2B, we present them as a function of *ϵ*_*inter*_. The results elucidate that < *R*_70*A*−70*B*_ > and < *R*_70*B*−*trimer*_ > increase with decreasing *ϵ*_*inter*_, favouring distributed domain architecture in apo hRPA when inter-domain interaction is weak. For instance, < *R*_70*A*−70*B*_ > and < *R*_70*B*−*trimer*_ > at *ϵ*_*inter*_ = 0.3 kcal/mol are found to be 36.2 ± 6.1 Å and 59.5 ± 13.6 Å, which are ∼ 1.3 and ∼ 1.4 times higher than the same observed at *ϵ*_*inter*_ = 1.0 kcal/mol. Moreover, we examined the distributions of *R*_70*A*−70*B*_ and *R*_70*B*−*trimer*_ at different *ϵ*_*inter*_ and presented them in the insets of Fig 2A and 2B. *R*_70*A*−70*B*_ and *R*_70*B*−*trimer*_ at *ϵ*_*inter*_ = 0.3 kcal/mol vary within ranges 25–45 Å and 38–78 Å respectively. The distributions cover the same ranges of inter-domain distances as observed in an all-atom simulation [41] of apo hRPA at 300 K (shown by shaded region). Furthermore, to confirm if *ϵ*_*inter*_ = 0.3 kcal/mol is a good measure of the inter-domain interaction strength to mimic the domain-domain flexibility and inter-domain dynamics of hRPA in apo state, we examined the compactness of the molecule by measuring its radius of gyration (*R*_*g*_) and presented the result in Fig 2C. We found an excellent match between the reported range (shaded region) of *ϵ*_*inter*_ obtained from an atomistic simulation and our coarse-grained simulations [41]. A SAXS experiment involving an apo hRPA at 288 K temperature [41] suggests that $<R_{g}^{expt}>$ ∼ 38.8 Å which is very similar to $<R_{g}^{sim}>$ ∼38.9± 4.1 Å as obtained from our simulation. The agreement between our coarse-grained results, and the atomistic simulation and experimental results thus, suggests that *ϵ*_*inter*_ = 0.3 kcal/mol correctly models the inter-domain contact strength in apo hRPA and efficiently scans the relevant ensemble of conformations encompassing its complete structural diversity. For the rest of the study, we, therefore, adopted *ϵ*_*inter*_ = 0.3 kcal/mol to model the conformational heterogeneity of hRPA and investigate its association with the ssDNA substrate. Henceforth we referred this hRPA model as a flexible hRPA model. As a controlled simulation with restricted hRPA domain movements (*ϵ*_*inter*_ = 1.0 kcal/mol), the state is referred as the rigid hRPA model in the rest of the study. Before proceeding further to unravel the role of inter-domain flexibility of hRPA in its association dynamics with ssDNA, we estimated the correlation between inter-domain fluctuations at each step using dynamic cross-correlation analysis. Detail about the calculation of dynamic cross-correlation using the Normal Mode Analysis is provided in Materials and Methods (see “Normal Mode Analysis” subsection). The results corresponding to flexible and rigid hRPA models are presented in Fig 2D and 2E respectively. The higher cross-correlation observed at low inter-domain strength indicates strong domain-domain interactions and connected movements of hRPA domains (correlated/anti-correlated domain movements). The behaviour is less prominent for the rigid hRPA system, i.e. with higher inter-domain strength (Fig 2E). A careful comparison of both plots extricates further details about the inter-domain crosstalk/interactions. Our results suggest DBDs A & C, B & D and B & E exhibit a strong correlated movement whereas DBDs A & D show a strong anti-correlated movement among the non-adjacent DBD pairs in the flexible hRPA system. Notably, such prominent correlated domain movements are not observed when the inter-domain interactions are strong as found in the rigid hRPA model.

### Kinetic and thermodynamics study of hRPA-ssDNA binding unravel existence of binding intermediates

**Fig 3:**
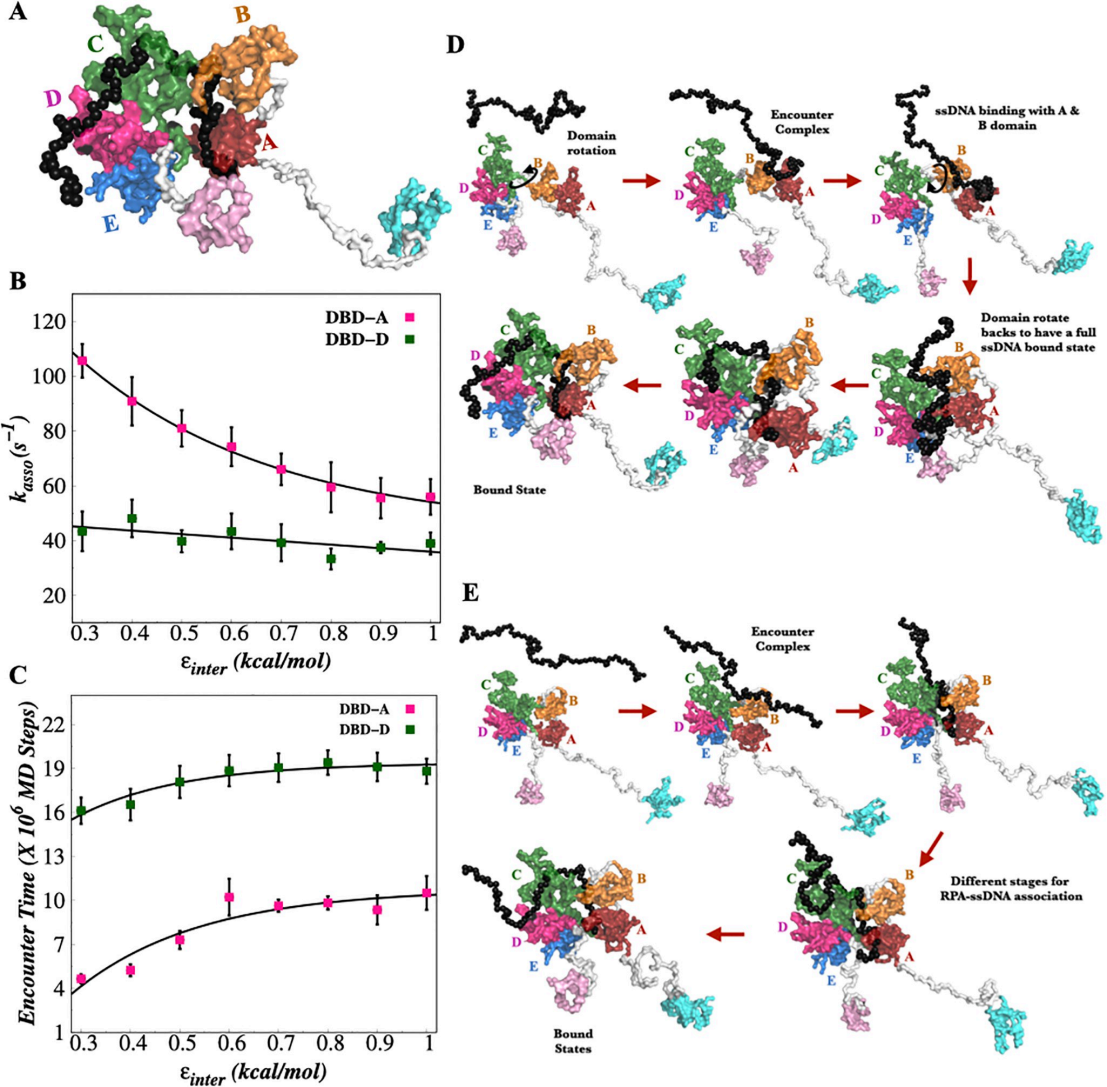
Kinetic of hRPA-ssDNA binding. (A) The bound structure of hRPA-ssDNA complex for flexible hRPA system. (B) The association rate of ssDNA to DBD-A (pink color) and DBD-D (green color) of hRPA as a function of *ϵ*_*inter*_. (C) Encounter time for the formation of first specific contact between hRPA’s respective domain (DBD-A, pink color and DBD-D, green color) and ssDNA nucleotides as a function of *ϵ*_*inter*_. (D) The snapshots of ssDNA with flexible hRPA system (*ϵ*_*inter*_ = 0.3 kcal/mol) in the unbound state, during the formation of hRPA-ssDNA complex and final bound state conformation. (E) The snapshots of ssDNA with rigid hRPA system (*ϵ*_*inter*_ = 1.0 kcal/mol) in the unbound state, during the formation of hRPA-ssDNA complex and final bound state conformation.

Do the correlated and anti-correlated movements of hRPA domains regulate the interactions with ssDNA and if yes, how? To understand, it is necessary to investigate first if the domain flexibility at all contributes in the association with ssDNA substrate. We performed molecular simulations of hRPA with 60 nt ssDNA to test this, starting from the unbound configurations for both flexible and rigid hRPA models and estimated the binding kinetics. By comparing the crystal structure of *U. maydis* RPA and ssDNA (PDB ID: 4GNX & 4GOP) [37], hRPA (AB domains)-ssDNA bound crystal structure (PDB ID: 1JMC) [31] and the previously captured bound state from the simulation study of *U. maydis* RPA with different lengths of ssDNA intermediate starting from an unbound state of *U. maydis* RPA and ssDNA [26], we confirmed the correct bound state of hRPA-ssDNA. Fig 3A represents the hRPA bound ssDNA configurations for flexible hRPA system and hRPA-ssDNA conformations at other *ϵ*_*inter*_ values are shown in S2 Fig. Each DBD has varying footprints on ssDNA. We tracked the formation of specific interfacial contacts between hRPA and ssDNA to monitor the progression of their binding. Interfacial contacts between the two were identified if two non-hydrogenous atoms in the all-atom structure of the hRPA-ssDNA complex, each from an amino acid of hRPA and a nucleotide of ssDNA, are within a distance of 3.7 Å. To find out the interfacial contacts involving DBD-A and B, we analyzed the all-atom structure of the hRPA(AB)-dC8 (PDB ID: 1JMC) [31] complex. The interfacial contacts in trimer core—ssDNA is obtained from the superimposed all-atom structure of trimer core (PDB ID: 1L1O) [34] on the template of *U. maydis* RPA-ssDNA crystal structure (PDB ID: 4GOP) [37]. We found 58 interfacial contacts (16 charged, 13 aromatic, 10 polar, and 19 hydrophobic residues) spanning over the DBDs A (9 contacts), B (11 contacts), C (21 contacts) and D (17 contacts). The rates of ssDNA association were measured at the distantly positioned DBDs A and D of the hRPA by measuring the time required to form at least 80% of the recognized interfacial hRPA-ssDNA contacts. Our results presented in Fig 3B exhibit association rates of ssDNA at DBD-A and DBD-D as a function of inter-domain contact strength (*ϵ*_*inter*_). Our results suggest that *k*_*asso*_ of ssDNA at DBD-D has significantly less dependency on *ϵ*_*inter*_ compared to that at DBD-A. At DBD-A, the rate of association with ssDNA decreases approximately 2 times following an exponential decay with the increase in *ϵ*_*inter*_. The kinetic results imply that the association of ssDNA to hRPA is facilitated from the DBD-A end where the hRPA domains are more flexible and dynamic to be engaged in crosstalk with each other. To understand how does the dynamics of hRPA domains facilitate the binding of ssDNA, we further measured the encounter time between hRPA and ssDNA at all inter-domain strengths. The encounter time is estimated from the time required for the formation of first specific contact between hRPA’s respective domain and ssDNA nucleotides. Our result in Fig 3C shows that high conformational flexibility of DBD-A of hRPA (at low *ϵ*_*inter*_) favours a faster anchoring of the ssDNA on hRPA, confirmed by the respective short encounter time. We find that the encounter time at DBD-A increases significantly as *ϵ*_*inter*_ increases from 0.3 to 0.6 (kcal/mol) and saturates for *ϵ*_*inter*_ > 0.6 kcal/mol as shown in Fig 3C. This indicates that the hRPA domains that are flexible (such as DBD-A and B) interact with each other more diversely with increasing freedom in domain movement (with lower *ϵ*_*inter*_ values) and assume the most favourable orientation to facilitate association with ssDNA, as evident from the shorter encounter time and the faster association kinetics. DBDs that are less dynamic such as DBD-D, which is involved in trimer core formation and hence is conformationally more rigid than DBD-A, shows roughly constant time (Fig 3C) in encountering the ssDNA, indicating its limited role in regulating the overall hRPA-ssDNA association kinetics. The snapshots of the hRPA system before, during and after the protein-ssDNA complex are shown in Fig 3D and 3E for the flexible and rigid hRPA system respectively. S1 and S2 Movies illustrate the binding mechanism of ssDNA with flexible and rigid hRPA system respectively. To this end, one should note that a larger inter-domain flexibility does not necessarily correspond to faster association kinetics. To test whether a further reduction in *ϵ*_*inter*_ (high interdomain flexibility) favors the hRPA and ssDNA association, we estimated two parameters namely, *τ*_*encounter*_, defined as the time required to establish the first non-specific short-ranged interaction (encounter) between ssDNA and hRPA residues (if any *C*_*α*_ residue of DBD-A and phosphate bead of ssDNA are at a distance less than 10 Å from each other in our CG structures), and *τ*_*evolution*_, which represents the time required for the non-specific ssDNA-hRPA complex to evolve as the specific ssDNA-hRPA complex for each *ϵ*_*inter*_ including *ϵ*_*inter*_ < 0.3 kcal/mol (S3 Fig). Our results clearly suggest that while increasing the domain flexibility enhances the encounter possibility between hRPA and ssDNA residues, the evolution time (*τ*_*evolution*_) describes an opposite trend. This is because the enhanced flexibility hinders the formation of specific contacts resulting in the escape of the complex to a non-specific state instead of the desired specific hRPA-ssDNA complex. The kinetics of RPA-ssDNA specific complex formation is therefore a tradeoff between the two and our result suggests that the fastest kinetics is ensured at *ϵ*_*inter*_ ∼ 0.3 kcal/mol, where both the rates (*τ*_*encounter*_ and *τ*_*evolution*_) are optimized and it is already mentioned that at this inter-domain contact strength, the conformational heterogeneity exhibited by the model protein matches with the previously characterized apo-state conformation of hRPA [41].

**Fig 4:**
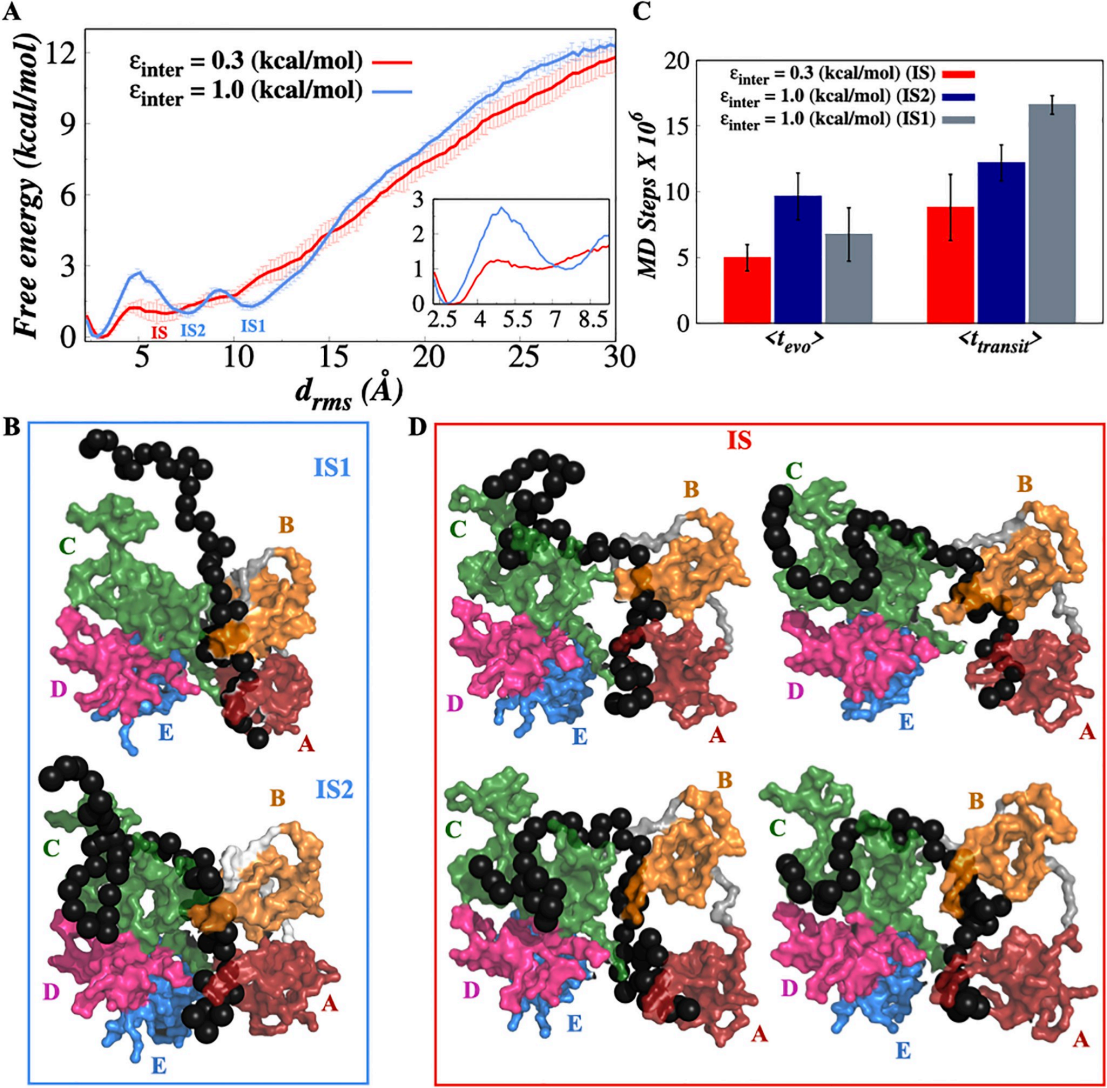
Thermodynamics of hRPA-ssDNA binding. (A) 1D free energy landscape of hRPA-ssDNA binding as a function of the distance root-mean-square deviation of hRPA-ssDNA binding distances *d*_*rms*_, between the hRPA and ssDNA beads that are in contact for flexible (red colored) and rigid hRPA model (blue colored). The inset at the bottom right is a magnified view of the binding free energy landscape focusing on the transition between the bound state and the intermediate states nearest to it. The error analysis was done by performing three independent umbrella sampling simulations. Error bars are omitted from this inset for clarity. (B) The associated structures of the intermediate states (IS1 and IS2) obtained for the rigid hRPA model at the *d*_*rms*_ value of 10–12 Å and 7–9 Å. (C) Evolution time between the encounter—intermediate states and the transition time between the bound—intermediate states for flexible (*ϵ*_*inter*_ = 0.3 kcal/mol) and rigid hRPA model (*ϵ*_*inter*_ = 1.0 kcal/mol). (D) The associated structures of the intermediate states obtained for flexible hRPA model at *d*_*rms*_ value of 5.5–7.5 Å.

We next turned to investigate the thermodynamics of hRPA-ssDNA binding. The consensus view is that hRPA engages ssDNA in three stages namely, initial, intermediate and final stages although the intermediate binding mode remained poorly characterized [18, 41]. Exploring the free energy landscape of binding is an effective way to untangle the hidden layers of complexity in binding dynamics. Accordingly, we performed umbrella sampling simulations to calculate the free energy landscape of hRPA-ssDNA binding for both flexible and rigid hRPA models (details are presented in supporting information). The root-mean-squared deviation (*d*_*rms*_) of the distances between the hRPA and ssDNA beads that are in contact is used as the reaction coordinate. We emphasize that these contacts are different from that of the interfacial contacts (mentioned previously) and they serve different purposes as well. The interfacial contacts, characterised from the atomistic description of the bound state are essential to precisely recognize the amino acids of hRPA that participate in forming specific contacts only in the hRPA-ssDNA bound complex. Formation of the specific contacts do not necessarily capture the transient interactions and detailed dynamics of an overall binding process. Hence, we identified all those contacts between *C*_*α*_ residue of hRPA and phosphate bead of ssDNA that are less than 10 Å from each other in our CG structures. The choice of cut off distance is in accordance with the selection of cut off distance for different short range interactions (hydrogen bonding and aromatic interactions) used in our model. A distance greater than 10 Å therefore indicates that the ssDNA beads are off the hRPA protein surface. We note that similar cut off distance was chosen in previous studies as well [25, 26] to recognize the short range interactions between protein and ssDNA beads in a coarse grained protein-ssDNA description of comparable resolution. Using this cut off distance, we identified ∼ 160 common contacts between hRPA and ssDNA beads by analysing the ensemble of bound state complexes explored in our simulations. The binding free energy profiles as a function of *d*_*rms*_ are presented in Fig 4A for both flexible and rigid hRPA models. A high *d*_*rms*_ value indicates an unbound state whereas reduction in the *d*_*rms*_ indicates progression of the binding event, resulting into a stable hRPA-ssDNA bound complex denoted by a small *d*_*rms*_ (< 3 Å) value. Our result elucidates a binding free energy profile featuring multiple intermediate states for hRPA molecule with rigid domain architecture (*ϵ*_*inter*_ = 1.0 kcal/mol). The unbound states (US) can be characterized by *d*_*rms*_ > 30 Å that features a significantly high free energy. The binding initiates when hRPA domains first encounter the ssDNA substrate and encounter complex (EC) forms at *d*_*rms*_ ∼ 28–30 Å. With further progression in binding, two distinct intermediate states can be noticed corresponding to *d*_*rms*_ ∼ 11.0 Å (IS1) and 7.5 Å (IS2) respectively. A free energy barrier of 0.7 kcal/mol separates the two intermediate states. The associated configuration suggests that DBDs A and B are engaged with approximately 23–26 nt ssDNA while forming the IS1 (see Fig 4B). The IS2 encompasses DBD-C in addition to A and B domains to engage with ∼ 40 nt ssDNA substrate. The bound states (BS), which is stabilized by 0.97 kcal/mol compared to the IS2 state, appears at *d*_*rms*_ ∼ 2.5 Å and involve four DBDs A-D engaged with ∼ 50 nt ssDNA. It should be noted that all these ∼ 50 nucleotides do not form specific contacts simultaneously with the protein residues to form the bound state. To identify the nucleotides that were in contact with the protein in most of the bound state complexes, we estimated the relative occurrence of each nucleotide in the ensemble of hRPA-ssDNA bound states. S4 Fig clearly shows that primarily 35–40 nts are required to cover all the hRPA domains and one end of the ssDNA remains flanking (as can be seen in some of the bound states in S3 Fig). The flanking region may get into contact with hRPA only transiently increasing the total number of engaging nucleotides with hRPA in the bound state. The binding free energy profile of flexible hRPA (corresponding to *ϵ*_*inter*_ = 0.3 kcal/mol) demonstrate significant differences. The profile shown in Fig 4A (red colored) differs from that of the rigid hRPA model (blue colored) in at least two aspects: (1) The initial binding up to *d*_*rms*_ ∼ 8 Å features a downhill, comparatively lower free energy binding profile compared to that obtained at *ϵ*_*inter*_ = 1.0 kcal/mol, indicating the formation of intermediate states engaging DBDs A-C are facilitated compared to that in hRPA with rigid domain architecture. (2) A broad and shallow minimum is observed corresponding to *d*_*rms*_ ranging between 5–8 Å, which indicates the ensemble of intermediate states featuring the same free energy instead of a single pronounced intermediate conformation (IS2) that precedes the final bound hRPA-ssDNA complex in rigid hRPA model. The impact of such downhill binding free energy profile on the kinetics of association is found to be two-pronged. Firstly, we estimated the average time taken by the unbound state to evolve into the intermediate complex (< *t*_*evo*_ >) for both flexible and rigid hRPA systems. < *t*_*evo*_ > for flexible hRPA is approximately two times faster (see Fig 4C) than when the domains of hRPA are rigid (*ϵ*_*inter*_ = 1.0 kcal/mol). Secondly, the bound state is separated by a free energy barrier of merely 0.25 kcal/mol from the intermediate state for flexible hRPA system compared to 1.781 kcal/mol for rigid hRPA system, explaining the origin of speedup in the average transition time from the intermediate state to the bound state (< *t*_*transit*_ >) in flexible hRPA compared to rigid hRPA model. The associated structures of the intermediate states obtained for flexible hRPA model (*ϵ*_*inter*_ = 0.3 kcal/mol) reveal that configurationally they differ (shown in Fig 4D) but are approximately degenerate free-energetically which allows rapid interconversion among them, explaining why it is difficult to detect and characterize them experimentally. Notably, a study based on atomistic simulation [41] has failed to capture any distinct intermediate state while studying hRPA-ssDNA association from a restrained starting configuration that maintained the canonical base-stacking interactions between aromatic residues and DNA bases as found in the initial X-ray crystal structures. We note that the strategy had limited opportunity in sampling the ssDNA-hRPA conformational space due to biased staring configuration that possibly resulted in missing the intermediate state ensemble as observed by us. On contrary, our results obtained from an unbiased coarse-grained simulation of apo hRPA and ssDNA substrate establish the consensus view that hRPA engages ssDNA in initial, intermediate and final stages and characterize the intermediate states for the first time to the best of our knowledge.

### Mechanism of hRPA-ssDNA binding: Role of hRPA domain dynamics

**Fig 5:**
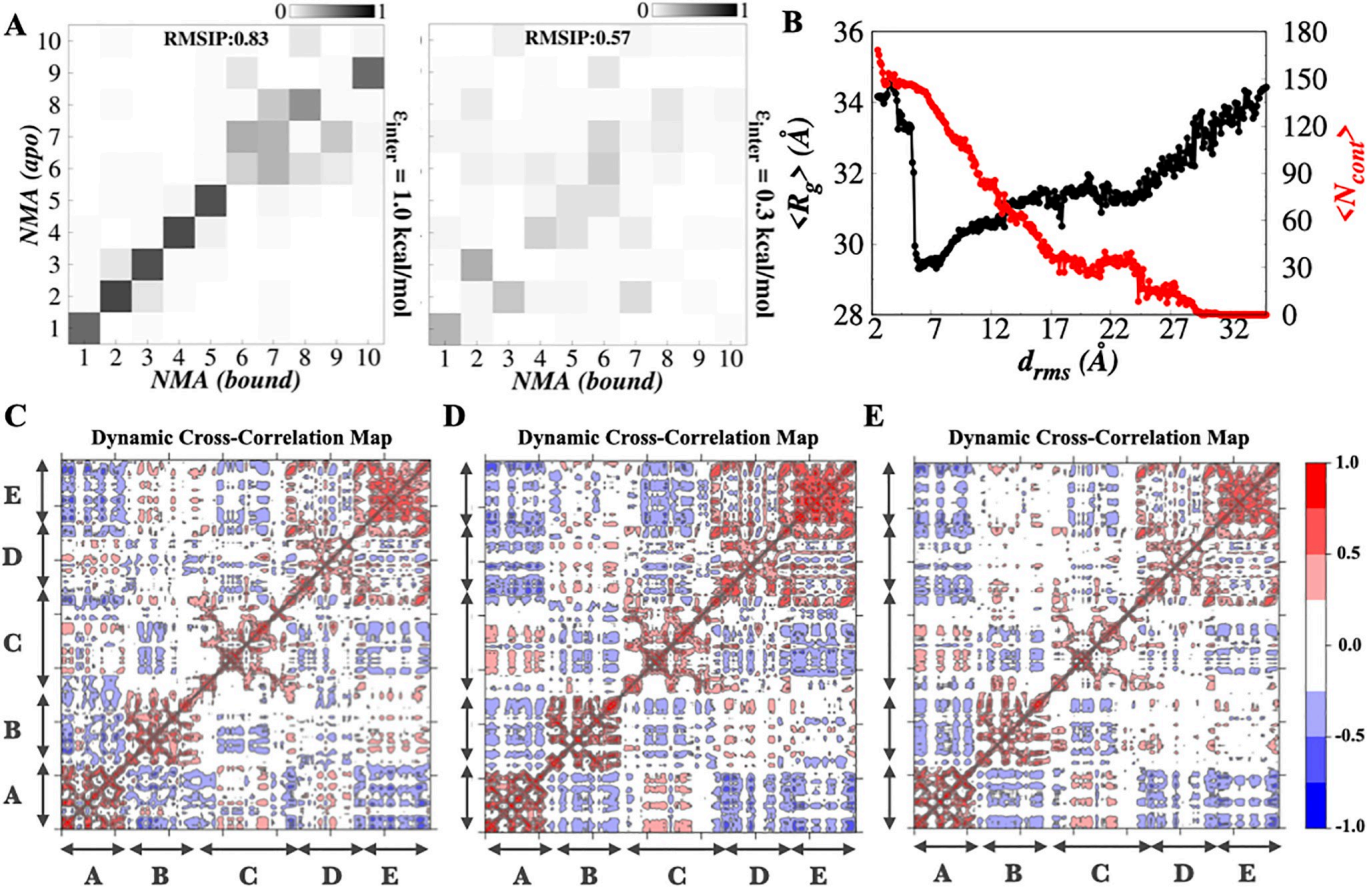
Normal Mode Analysis of apo hRPA and hRPA-ssDNA complex. (A) The root-mean-square inner product (RMSIP) of the two NMA subsets (NMA of apo hRPA and ssDNA bound hRPA state) for rigid hRPA model (*ϵ*_*inter*_ = 1.0 kcal/mol) (left panel) and flexible hRPA model (*ϵ*_*inter*_ = 0.3 kcal/mol) (right panel). RMSIP value of 1 indicates the identical directionality in both states. (B) The average radius of gyration of hRPA < *R*_*g*_ > (black color) and the average number of interfacial contacts < *N*_*cont*_ > (red color) formed as a function of *d*_*rms*_ for flexible hRPA model. (C-E) The dynamic cross correlation maps (DCCM) of (C) encounter complex, (D) intermediate state and (E) bound state of the ssDNA bound hRPA system averaged over all modes for the correlation between inter-domain fluctuations in the flexible hRPA model for the ssDNA binding core domains. The ssDNA binding domain regions are labeled and shown by the two headed arrow.

How do the hRPA domains function in the ssDNA processing mechanism? Do they engage synergistically or in a sequential fashion while interacting with ssDNA? How does the domain-domain crosstalk in apo hRPA influence its binding with ssDNA? To answer these questions, we calculated the normal modes of hRPA in the apo hRPA (NMA(apo)) and the hRPA-ssDNA complex (NMA(complex)) for flexible (S5 and S6 Figs respectively) and rigid system (S7 and S8 Figs respectively) separately and compare their similarities by estimating the root-mean-square inner product (RMSIP) of the normal modes. A higher value of RMSIP signifies similar normal modes and domain movements operating in both the apo and complex states. Alternatively, a lower RMSIP value suggests the protein undergoes a different set of conformational transitions while forming a complex with ssDNA than in its apo state. Our estimation of RMSIP from the calculated normal modes (including the first 10 normal modes each from the apo hRPA and bound state) for rigid and flexible hRPA models are presented in Fig 5A left and right panel respectively. The results suggest a higher RMSIP value (0.83) for the rigid hRPA model, denoting a similar dynamics of hRPA domains observed in both the apo conformation and the ssDNA bound complex (see Fig 5A). In contrast, a significantly lower value (RMSIP = 0.57) for the flexible hRPA model denotes a different dynamics of hRPA domains is operative in the apo state compared to its ssDNA bound complex (see Fig 5A). To gain further insight into how the movement of hRPA domains in the presence of ssDNA differ compared to its apo state, we characterized the complete binding pathway for the flexible hRPA model. This is done by simultaneously monitoring the overall compactness of the hRPA by estimating its < *R*_*g*_ > and the average number of contacts < *N*_*cont*_ > formed as a function of *d*_*rms*_ in the MD simulation trajectory. The results shown in Fig 5B demonstrate an initial reduction in the < *R*_*g*_ > with the decrease in *d*_*rms*_ until the binding leads to the formation of the intermediate state at *d*_*rms*_ ∼ 5–8 Å. The corresponding < *N*_*cont*_ > profile suggests that the encounter complex (when first contact forms) features a high < *R*_*g*_ > (∼ 32–35 Å) compared to its intermediate state. The associated structural representations portray gradual compaction of the hRPA domains until the intermediate state is formed. With further lowering of the *d*_*rms*_ the intermediate state transforms into the hRPA-ssDNA bound state by engaging the DBD-D but featuring a sharp enhancement in the < *R*_*g*_ >. The abrupt increase in < *R*_*g*_ > by ∼ 5 Å indicates that the binding of ssDNA at different hRPA domains are not independent events, rather inter-domain crosstalk plays an important role. To verify the same, we analyzed the dynamic cross-correlation (DCCM) between the domains using Normal Mode Analysis and present in Fig 5C–5E for the encounter complex, the intermediate state and the hRPA-ssDNA bound complex respectively, which were identified based on their respective *d*_*rms*_ values from the independent binding MD simulation trajectories. The analysis was performed for multiple encounter, intermediate and hRPA-ssDNA bound state complexes. The results illustrate the domain-domain crosstalk and the inter-dependency of their movement that provide two major insights: (i) The DCCM plot for apo hRPA presented in Fig 2D is noticeably different from the DCCM plots in Fig 5C–5E analyzed during the binding events. The major difference is in terms of the intensity that reflects the extent of domain-domain crosstalk. As the binding progresses, the reducing intensity of the domain-domain interaction pattern exhibits reduced interactions/crosstalks, reflecting binding induced rigidity in the hRPA domains. The domains engage with the ssDNA to result in a stable bound state at an entropic cost that comes from the increasing rigidity of the hRPA domains. (ii) The directionality in the domain movement changes remarkably along the binding pathway. For instance, DBD-A and DBD-D move concertedly whereas DBD-B and DBD-D display an anti-correlated movement in the encounter complex. Interestingly, the trends reverse entirely in the intermediate and bound state complexes (see Fig 5D and 5E).

**Fig 6:**
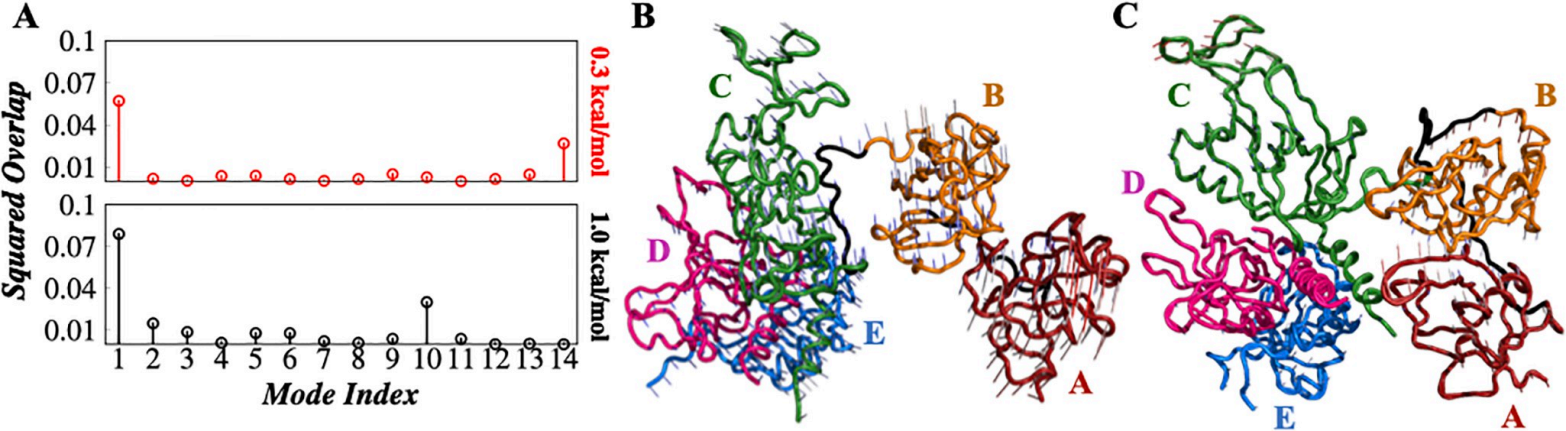
Role of hRPA domain dynamics. (A) The squared overlap analysis between the normal modes of hRPA protein and conformational difference vector between hRPA’s structure at the initial state (the unbound state of hRPA-ssDNA system) and the final state (the bound state of hRPA-ssDNA system) from the molecular dynamics simulation trajectory are shown for flexible hRPA model (*ϵ*_*inter*_ = 0.3 kcal/mol) (upper panel) and rigid hRPA model (*ϵ*_*inter*_ = 1.0 kcal/mol) (lower panel). Only nontrivial modes are shown. The high value of squared overlap represents the modes that contribute significantly to the conformational changes observed during the binding pathway. The vector field representation of the highest squared overlap valued non-trivial modes (mode 1) for (B) flexible hRPA model and (C) rigid hRPA model. The domains and linkers are color coded to match the domains as shown in Fig 1 top panel.

To probe the reversal in domain movements, we measured the squared overlap between the nontrivial normal modes of the hRPA and the conformational difference vectors between the encounter complex and the hRPA-ssDNA bound state sampled in our simulations (Fig 6A upper panel and Fig 6A lower panel for flexible and rigid hRPA model respectively). Squared overlap values identify the modes that contribute significantly to the conformational changes observed during the binding pathway. We are interested to identify the modes which contribute to the conformational changes observed during hRPA-ssDNA binding in flexible and rigid hRPA systems on the basis of squared overlap values [76]. For instance, the first nontrivial mode (mode 1) exhibits high squared overlap with the conformational difference vector for both flexible and rigid hRPA systems (Fig 6A). Additionally, the 14^*t*h^ non-trivial mode in the flexible hRPA and the 10^*th*^ non-trivial mode in the rigid hRPA system contribute significantly (Fig 6A). The movement of the first non-trivial modes for flexible and rigid hRPA model is shown in Fig 6B and 6C respectively (S3 and S4 Movies respectively). The vector field representation of these modes is used to clarify the differences in the magnitude and direction of the movement of hRPA domains. The larger arrows (vectors) in a domain indicates higher movement of that domain and vice versa. A comparative analysis of the same for flexible and rigid hRPA model indicates the higher movement of the domains in flexible hRPA (Fig 6B) than that of the rigid hRPA system (Fig 6C). To quantify the movement of these first nontrivial modes, we estimated the distances between DBD-B and trimer core, *R*_70*B*−*trimer*_ (Å) and between DBD-B and DBD-C, *R*_70*B*−70*C*_ (Å) as a function of the rotation around an imaginary axis connecting DBDs B and C (dihedral angle formed between the ABCD domains) Φ_*ABCD*_ and present them in S9 Fig for flexible and rigid hRPA systems. The parameters involve DBDs A-D as these four domains only engage in interacting with the ssDNA substrate. Our results point out towards the fact that both *R*_70*B*−*trimer*_ and *R*_70*B*−70*C*_ increase as a function of Φ_*ABCD*_ in hRPA with flexible domain dynamics. The same in rigid hRPA exhibit an anti-correlated inter-dependency with Φ_*ABCD*_. Mechanistically this means that when hRPA domains are dynamic as noted experimentally in its apo state, the highly dynamic B and A domain moves with respect to the trimer core. Such a movement around the connecting axis between DBDs B and C is best captured by the dihedral rotation Φ_*ABCD*_, which results in significant opening up of the high affinity A & B domain. This change is further supported by ∼ 4 Å change in the distance between DBD-A and DBD-C (*R*_70*A*−70*C*_) for flexible hRPA system as shown in S9 Fig. This plays a crucial role in facilitating ssDNA binding as DBD-A, which is a terminal domain, can easily face towards the ssDNA bases and mediate nonspecific interactions. But, DBD-B, that is positioned in between DBD-A and trimer core, rotates upside only when ssDNA binding induces rotation to enhance the binding interface with ssDNA substrate. This is confirmed by the simultaneous increase of *R*_70*B*−*trimer*_ and *R*_70*B*−70*C*_ distances (S9 Fig). We note that for flexible hRPA ssDNA binding angular movement of the protein domains is predominant over linear displacement. While the distance between DBD-B and DBD-D (*R*_70*B*−32*D*_) changes merely ∼1 Å (S9 Fig) in a flexible hRPA system compared to ∼2 Å (S9 Fig) in rigid hRPA ssDNA binding, the rotation around an imaginary axis connecting DBD-B and DBD-D (Φ_*ABDC*_) in flexible hRPA is twice compared to that in rigid hRPA-ssDNA complex (S9 Fig). Accordingly, DBD-B and DBD-D exhibit anti-correlated dynamics, separating away from each other in the DCCM plot of encounter complex. This continues until the intermediate state is reached after which the inter-domain interactions in Fig 5D and 5E follow the DCCM pattern of apo state of flexible hRPA but with reduced domain flexibility. On contrary, in the rigid hRPA model, where the movement of hRPA domains is restricted, the A and B domain does not undergo conformational changes suitable for ssDNA association, rather DBD-C inclines towards DBD-B and engages in interacting to selectively exclude its interaction with ssDNA. Another critical observation is that the protein domains in hRPA, when modeled flexibly, explore a greater range of the dihedral angles (S9 Fig) compared to when their dynamics are restricted. However, the corresponding ranges for *R*_70*B*−*trimer*_ and *R*_70*B*−70*C*_ are approximately two times more in rigid hRPA than in flexible hRPA system. Such rotation (around DBDs B and C) induced smaller changes in the hRPA architecture involving DBDs B and C is a signature of ssDNA binding induced changes in the hRPA conformation. The associated domain movements are minimal and different compared to that in apo state of hRPA molecule (also evident from Fig 5A) in order to minimize the associated free energy cost. On contrary, a large change in *R*_70*B*−*trimer*_ and *R*_70*B*−70*C*_ denote a distinct transition of rigid hRPA conformations during its binding with ssDNA. The difference in conformational dynamics suggest that the flexibility of hRPA domains allows it to follow a binding induced conformational transition that results in a smooth, downhill free energy pathway. The absence of any high free energy barrier in the associated binding free energy profile ensures faster binding kinetics. However, a rigid domain architecture of hRPA decouples its conformational transition from its binding with ssDNA, leading to a rugged binding free energy landscape with distinct minimum for the intermediate states. At the end, it should be noted that 10^*th*^ and 14^*th*^ non-trivial modes in the rigid and flexible hRPA system respectively do not have noticeable impact on the conformational changes of hRPA during its binding with ssDNA. We tested the same by measuring all the distances and rotational angles and did not notice any significant domain movements. Therefore, the results are not shown.

**Fig 7:**
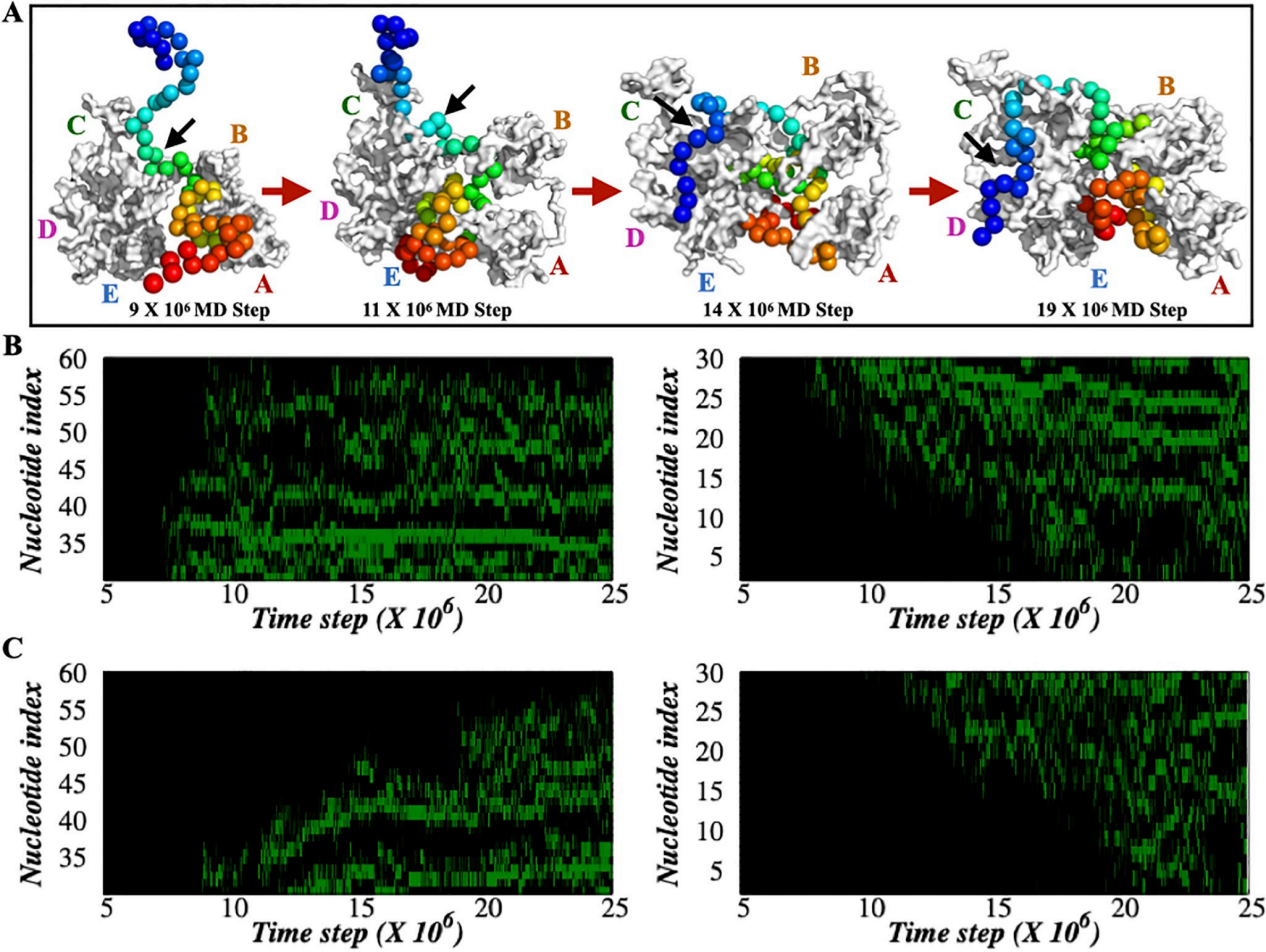
Characterization of ssDNA bulges on the hRPA protein surface. (A) The snapshots sampled at different MD steps represent the formation of bulges by ssDNA during its association with hRPA. (B, C) Time evolution of the ssDNA nucleotide bulge index positioned at the protein surface is shown for ssDNA length binding with DBDs A-B (left panel) and with DBDs C-D (right panel) for (B) flexible hRPA system (*ϵ*_*inter*_ = 0.3 kcal/mol) and (C) rigid hRPA system (*ϵ*_*inter*_ = 1.0 kcal/mol). The changed/unchanged bulge index with the MD time steps reflects the formation of dynamic/static bulges formed. Bulges were quantified by selecting a distance criterion between the phosphate atoms of ssDNA which were less than 60% of its Kuhn length present in the stretched conformation.

Another way to track the hRPA-ssDNA binding event is to monitor the movement of each nucleotide on the hRPA surface beginning with the first non-specific contact with hRPA. Previous studies involved in probing the hRPA-ssDNA interactions [25, 26] have illustrated the formation of DNA bulges during their binding. The bulges in general are reported as both static and dynamic [25, 26, 78], and therefore, their roles in the binding of proteins remain unclear. Here, we monitored if the bulges are at all dynamic and how do they influence binding with hRPA. The DNA bulges on the hRPA surface are identified if the first (*i*^*th*^) and last (*j*^*th*^) phosphate group participating in a bulge is positioned within 10 Å from the hRPA surface and the distance (*r*_*ij*_) between the *i*^*th*^ and *j*^*th*^ phosphate atoms of ssDNA is less than at least by 60% to its Kuhn length in a stretched conformation. The snapshots presented in Fig 7A show the bulge formation by ssDNA during the association with hRPA. To explore the role of bulges in hRPA-ssDNA association, we studied the time evolution of phosphate groups that appear first when a bulge is formed. The results are presented in Fig 7B and 7C for flexible and rigid hRPA systems respectively. Green dots in the plots present indices of those phosphate groups in Fig 7B and 7C, representing the movement of bulges. The results reveal differences in bulge propagation between flexible and dynamic hRPA systems. In flexible hRPA (Fig 7B), ssDNA binds almost simultaneously to DBD-A and DBD-B domains with high affinity. Since DBD-A and DBD-B are connected by a long flexible linker, the almost simultaneous association of both the domains is feasible if it is induced by ssDNA binding. The dynamic DNA bulges promote stepwise progression of ssDNA (monitored by the change in bulge index with simulation time) that synergistically mediate contacts with hRPA domains as well as orient it suitably for further propagation. However, in the rigid hRPA system, the domain involvement (DBD-A & DBD-B) in binding with ssDNA is successive (monitored by the change in bulge index with simulation time) (Fig 7C). The involvement and movement of the nucleobases through bulges on the DBD-C and DBD-D domain are similar for both flexible and rigid hRPA systems, indicating the major differences in hRPA-ssDNA binding between flexible and rigid hRPA systems are due to the formation of the intermediate state, which is facilitated by the movement of protein domains in hRPA. Towards this end, it should be noted that the low surface tension in the CG model may also contribute in regulating the dynamics of the bulges.

## Discussion

The primary function of RPA is to protect ssDNA intermediates from nucleases and to act as a platform to recruit other proteins during crucial DNA metabolic processes. This particular protein and in general many other ssDNA binding proteins that are involved in DNA processing machinery are modular in nature with multiple constituting protein domains connected via flexible linkers. Accordingly, their architectures are best described in terms of ensembles of conformation. In fact, the conformational heterogeneity of hRPA has been realised experimentally in its apo state, although the complete protein conformation could not be resolved to date and only the structures of its individual constituting protein domains are determined. The lack of complete information regarding the architecture of the whole hRPA molecule impedes probing the mechanism of its association with ssDNA, which is essential to figure out how cells preserve the genomic integrity during DNA replication, recombination and base excision repair processes. In this study, we addressed the issue and probed the thermodynamics and kinetics of hRPA association with ssDNA using a state-of-the-art CG protein-DNA model. By carefully modulating the inter and intra-domain protein interactions, we mimicked the conformational heterogeneity of hRPA in its apo state as quantified by a SAXS and an all-atom simulation study. It should be noted that modulating the inter-domain contact strength is an ad hoc parameter and it is always more desirable to model inherent flexibility of any protein. However, the scope is limited within a CG framework. We, therefore, treated the inter-domain contact strength as a tunable parameter to model the inter-domain flexibility of a protein while focusing on its binding with ssDNA. It is worth mentioning that that while formulating a universal *ϵ*_*inter*_ is desirable for all those proteins that have multiple ssDNA binding domains, the same is practically unfeasible with this kind of simplistic model as each protein differs from the other in terms of their organization, area and sequence composition of inter-domain interfaces. The kinetic experiment using the modelled hRPA along with ssDNA suggests that inter-domain dynamics of the hRPA molecule favours the anchoring of the ssDNA to hRPA. A comparison with a controlled simulation featuring a conformationally rigid domain architecture of hRPA further reveals that the kinetic efficiency of ssDNA binding in hRPA with flexible domain architecture primarily stems from the side of DBDs A and B, which are more dynamic compared to DBDs C and D that participate in the formation of the largest trimer core subunit (hRPA70) of hRPA. The result is supported by a shorter time to encounter ssDNA at DBD-A compared to two times longer encounter times estimated at DBD-D. The thermodynamic study using the umbrella sampling method on the other hand unravels the binding free energy landscape of the hRPA molecule. The topology of the landscape is found to be dependent on the interactions and dynamics among the constituting protein domains of hRPA. A hRPA molecule with short-ranged domain movement results in a binding free energy landscape with two distinct, stable intermediates prior to forming the final bound complex with ssDNA. The structural characterization of the intermediates shows high affinity involvement of DBDs A and B followed by weak affinity association with the DBD-C. In contrast, the hRPA molecule with dynamic domains produces a nearly downhill binding free energy landscape while interacting with ssDNA. Interestingly, the landscape shows the formation of an ensemble of intermediate states (IS) instead of a distinct intermediate state as observed for hRPA domains with rigid architecture. We argue that the degeneracy in free energy of the ensemble of intermediate states make it difficult to precisely characterize intermediate states although it captures the structural features of initial and final states. The result underpins the consensus view that hRPA engages with ssDNA in initial, intermediate, and final stages although the intermediate binding mode remained poorly characterized [18, 41].

Our study further elucidates that the differences in the binding free energy landscapes of hRPA with flexible and rigid domain architecture are primarily due to their interactions/crosstalks. The hRPA model with rigid domain architecture exhibits limited domain movement which raises the free energy barrier when hRPA domains reorient to associate with ssDNA, resulting in distinct and free energetically stable intermediates in the binding free energy landscape. In comparison, in the flexible hRPA model, conformational flexibility of hRPA domains allows ssDNA to induce conformational transition in its domain organisation such that hRPA binds to ssDNA and reorients to achieve the final bound state conformation simultaneously. The binding induced conformational changes in hRPA and ssDNA substrate produces a downhill binding free energy landscape. Mechanistically, the reorientation of the hRPA domain involves rotation of DBD-B to open up its ssDNA binding patch, allowing it to associate with ssDNA synergistically with DBD-A which results in the rapid evolution of the encounter complex into the intermediate state. The domain-domain movement information of apo hRPA is useful in explaining the different movements of hRPA domains in its binding with ssDNA. The movement of DBD-B is anti-correlated to that of DBD-D in the encounter complex although they share correlated dynamics in the apo and the final bound states, confirming the role of ssDNA in inducing different domain dynamics in DBD-B. The anti-correlated domain movement leads to the formation of intermediate like states. A consistent molecular picture is observed while investigating the ssDNA-hRPA association by following ssDNA footprints. Our study suggests that ssDNA association to hRPA proceeds via DNA bulge formation, where a segment of outwardly positioned loop-like structure forms on ssDNA due to nuances in the protein-DNA interactions. The bulges are dynamic in nature and therefore, tracking the time evolution of the bulge position shades insight into the possible association mechanism. It should be noted that the low surface tension in the CG model may also contribute in regulating the dynamics of the bulges. The method confirms that when the hRPA domains are flexible and able to interact freely, the association of ssDNA begins almost simultaneously at DBD-A and DBD-B although the same happens in a sequential manner for the rigid architecture of hRPA domains, elucidating the molecular origin of comparatively faster binding kinetics in hRPA with flexible domain architecture. The result also indicates that the association of ssDNA with hRPA is kinetically largely governed by the dynamics of DBD-A and DBD-B, underscoring the significance of hRPA domain dynamics in its functions in DNA machinery. Indeed, it will be worthwhile to explore how the mutations in these two domains are linked with domain dynamics and thereby modulate the hRPA association with ssDNA intermediates.

To summarize, our study using the latest computational framework unravels the role of hRPA domain architecture in hRPA-ssDNA association, which is a crucial intermediate state in several DNA metabolic processes. While the consistency of our simulation results with several experimental observations is encouraging and sheds new light into the rich dynamics of hRPA binding to ssDNA, future experiments in combinations with our simulation study are expected to pave the way to resolve the functional details of hRPA molecules in various DNA processing machinery.

## Supporting information

S1 Fig Root-mean-squared-deviation (RMSD) plot of the hRPA’s individual resolved domains from our simulation trajectory as a function of MD step. (A) RMSD plot of Domain-F as a function of time. (B) RMSD plot of Domain-A as a function of time. (C) RMSD plot of Domain-B as a function of time. (D) RMSD plot of Domain-C as a function of time. (E) RMSD plot of Domain-D as a function of time. (F) RMSD plot of Domain-E as a function of time. (G) RMSD plot of Domain-WH as a function of time.
(TIFF)

S2 Fig The bound structures of hRPA-ssDNA at different interdomain strength (*ϵ*_*inter*_). The snapshots of hRPA-ssDNA bound state conformations are shown. The ssDNA binds with hRPA’s A, B, C and D domain at *ϵ*_*inter*_ of (A) 1.0 kcal/mol (B) 0.9 kcal/mol (C) 0.8 kcal/mol (D) 0.7 kcal/mol (E) 0.6 kcal/mol (F) 0.5 kcal/mol (G) 0.4 kcal/mol.
(TIFF)

S3 Fig The encounter time (*τ*_*encounter*_) for the non-specific short-ranged interaction and evolution time (*τ*_*evolution*_) for the evolution of non-specific complex to specific ssDNA-hRPA complex at different interdomain strength (*ϵ*_*inter*_). The encounter time, *τ*_*encounter*_, for the first non-specific contact formation between any *C*_*α*_ residue of the DBD-A and ssDNA nucleotide (non-specific short-ranged interaction) and the evolution time, *τ*_*evolution*_, for the non-specific complex to evolve as the specific hRPA-ssDNA complex.
(TIFF)

S4 Fig Relative occurrence of ssDNA nucleotides on hRPA protein surface. The relative occurrence of each nucleotide on hRPA surface in the ensemble of hRPA-ssDNA bound states as a function of DNA nucleotide.
(TIFF)

S5 Fig Normal Mode Analysis of apo hRPA protein in flexible hRPA model. Eigenvalues, frequencies and fluctuations of normal modes for apo hRPA at *ϵ*_*inter*_ = 0.3 kcal/mol. Fluctuations are calculated based on averages for all modes weighted by eigen values.
(TIFF)

S6 Fig Normal Mode Analysis of hRPA protein from hRPA-ssDNA complex in flexible hRPA model. Eigenvalues, frequencies and fluctuations of normal modes for hRPA-ssDNA complex at *ϵ*_*inter*_ = 0.3 kcal/mol. Fluctuations are calculated based on averages for all modes weighted by eigen values.
(TIFF)

S7 Fig Normal Mode Analysis of apo hRPA protein in rigid hRPA model. Eigenvalues, frequencies and fluctuations of normal modes for apo hRPA at *ϵ*_*inter*_ = 1.0 kcal/mol. Fluctuations are calculated based on averages for all modes weighted by eigen values.
(TIFF)

S8 Fig Normal Mode Analysis of hRPA protein from hRPA-ssDNA complex in rigid hRPA model. Eigenvalues, frequencies and fluctuations of normal modes for hRPA-ssDNA complex at *ϵ*_*inter*_ = 1.0 kcal/mol. Fluctuations are calculated based on averages for all modes weighted by eigen values.
(TIFF)

S9 Fig Quantitative analysis of the highest squared overlap valued non-trivial modes (mode 1) for flexible and rigid hRPA system. (A, B) The distances between DBD-B and trimer core, R70B-trimer (Å) (black color) and between DBD-B and DBD-C, R70B-70C (Å) (red color) as a function of the rotation around an imaginary axis connecting DBDs B and C (dihedral angle formed between the ABCD domains Φ_*ABCD*_) for (A) flexible hRPA model and (B) rigid hRPA model. (C, D) The distances between DBD-A and DBD-C, R70A-70C (Å) (black color) as a function Φ_*ABCD*_ for (C) flexible hRPA model and (D) rigid hRPA model. (E, F) The distances between DBD-B and DBD-D, R70B-32D (Å) (black color) as a function Φ_*ABDC*_ for (E) flexible hRPA model and (F) rigid hRPA model.
(TIFF)

S1 Table The parameters used to model protein. Mass and radius of Amino Acid Residues.
(DOCX)

S2 TableThe parameters used to model ssDNA.(A) Mass and radius of nucleotide components. (B) Table of equilibrium bond lengths (*r*^0^), bend angles (*θ*^0^) and torsion angles (*ϕ*^0^) in the coarse-grained DNA model. The phosphate and sugar of the adjacent site in the 5’ direction are denoted by P(5’) and S(5’) respectively. Similarly, the phosphate and sugar in the 3’ direction are denoted by P(3’) and S(3’) respectively. (C) Values of strengths *ϵ*_*ij*_ for base-stacking interaction in the 3SPN.2 DNA model. ↑ and ↓ denotes the sense and anti-sense strands respectively. 3’ ↑ and ↓ 5’ denotes adjacent bases in the 3’ and 5’ directions respectively. (D) The values of equilibrium distances (*σ*_*ij*_) and equilibrium angles ($\theta_{BS}^{0}$) for base stacking interactions. ↑ and ↓ denotes the sense and anti-sense strands respectively. 3’ ↑ and ↓ 5’ denotes adjacent bases in the 3’ and 5’ directions respectively.(DOCX)

S3 TableThe parameters used to model protein—ssDNA interactions.(A) The experimental values of strength $\epsilon_{ij}^{AA-B}$ for specific -*π*−*π* stacking interactions between different aromatic residue—nucleobase pairs (as reported by Rutledge *et al*.). The table values are adopted from the previous studies on the binding of proteins with ssDNA (references in the supplementary text). (B) The values of strength $\epsilon_{ij}^{Hydrogen}$ for hydrogen bonding interactions between non-aromatic *C*_*α*_ bead and nucleobase pairs.(DOCX)

S4 Table Protein-ssDNA model validation. The model has previously been used to capture the bound state conformations of a number of protein-ssDNA complexes (including SSB-ssDNA complex) irrespective of the length and sequences of ssDNA. A list of the PDB IDs, ssDNA sequence, Number of nucleotides and the RMSD value predicted from our model with respect to the relevant crystal structures is given.
(DOCX)

S5 Table The list of citations from where regarding the model parameters used for modeling of protein, ssDNA and protein-ssDNA interactions in this study. (DOCX)

S1 Movie The flexible hRPA (*ϵ*_*inter*_ = 0.3 kcal/mol) and ssDNA binding. The binding of ssDNA and flexible hRPA system. The protein domains are colored to match the domains as shown in Fig 1 top panel. The ssDNA is shown in ‘wheat’ color. For simplicity, only ssDNA backbone is shown in the movie.
(MP4)

S2 Movie The rigid hRPA (*ϵ*_*inter*_ = 1.0 kcal/mol) and ssDNA binding. The binding of ssDNA and rigid hRPA system. The protein domains are colored to match the domains as shown in Fig 1 top panel. The ssDNA is shown in ‘wheat’ color. For simplicity, only ssDNA backbone is shown in the movie.
(MP4)

S3 Movie First non-trivial of hRPA protein from hRPA-ssDNA complex in flexible hRPA model (*ϵ*_*inter*_ = 0.3 kcal/mol). The first non-trivial mode from hRPA-ssDNA complex in flexible hRPA model. The protein domains are colored to match the domains as shown in Fig 1 top panel.
(MP4)

S4 Movie First non-trivial of hRPA protein from hRPA-ssDNA complex in rigid hRPA model (*ϵ*_*inter*_ = 1.0 kcal/mol). The first non-trivial mode from hRPA-ssDNA complex in rigid hRPA model. The protein domains are colored to match the domains as shown in Fig 1 top panel.
(MP4)

S1 File References for supporting information text citations. (DOCX)
