# Supplementary figures and images for: Interdomain dynamics in human Replication Protein A regulates kinetics and thermodynamics of its binding to ssDNA
Source: PLoS One. 2023 Jan 19;18(1):e0278396. doi: 10.1371/journal.pone.0278396 (PMC9851514; doi:10.1371/journal.pone.0278396)

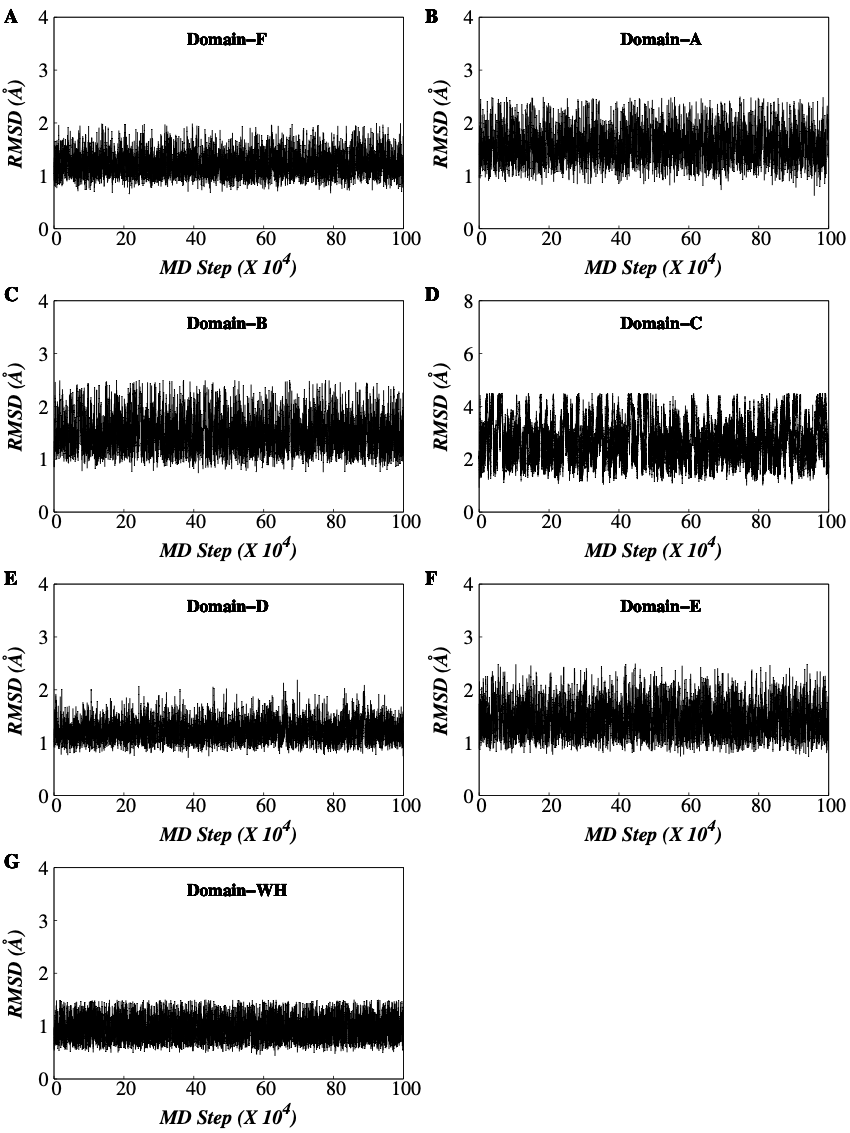

Supplement: S1 Fig — (A) RMSD plot of Domain-F as a function of time. (B) RMSD plot of Domain-A as a function of time. (C) RMSD plot of Domain-B as a function of time. (D) RMSD plot of Domain-C as a function of time. (E) RMSD plot of Domain-D as a function of time. (F) RMSD plot of Domain-E as a function of time. (G) RMSD plot of Domain-WH as a function of time. (TIFF) [file pone.0278396.s001.tiff]

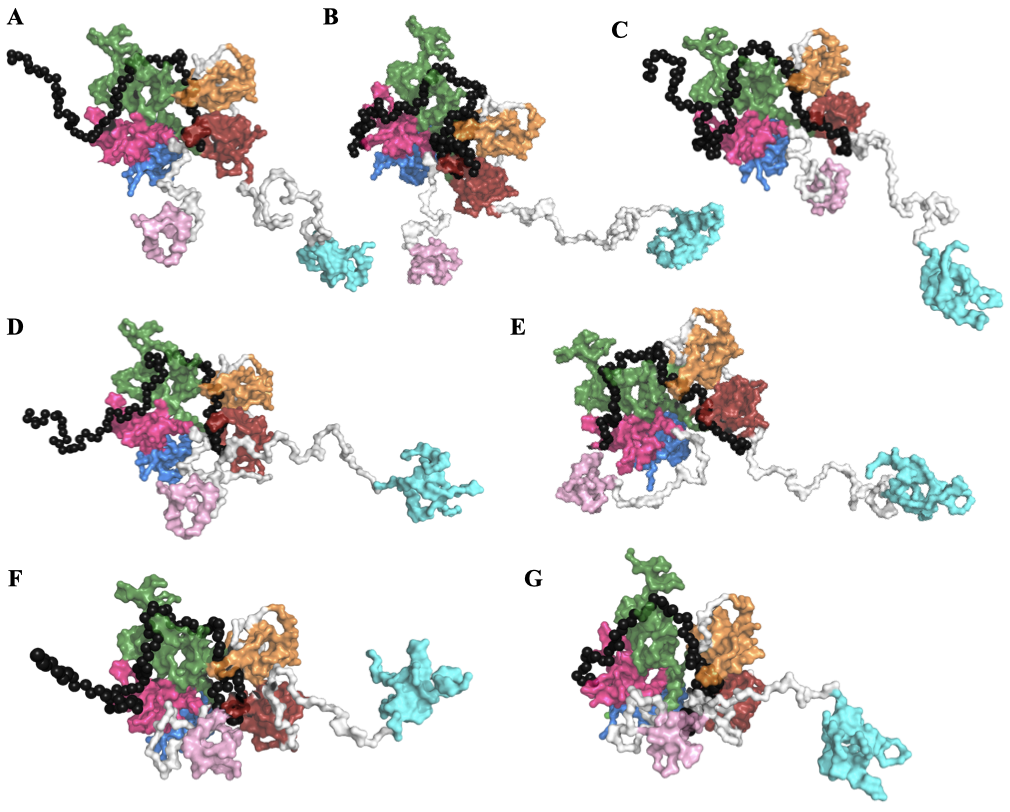

Supplement: S2 Fig — The snapshots of hRPA-ssDNA bound state conformations are shown. The ssDNA binds with hRPA’s A, B, C and D domain at ϵinter of (A) 1.0 kcal/mol (B) 0.9 kcal/mol (C) 0.8 kcal/mol (D) 0.7 kcal/mol (E) 0.6 kcal/mol (F) 0.5 kcal/mol (G) 0.4 kcal/mol. (TIFF) [file pone.0278396.s002.tiff]

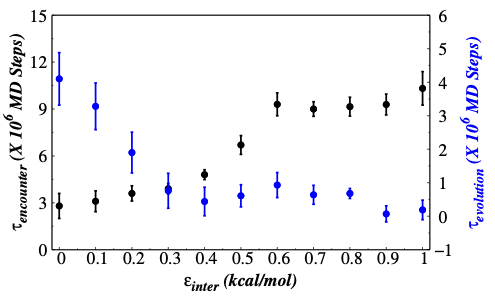

Supplement: S3 Fig — The encounter time, τencounter, for the first non-specific contact formation between any Cα residue of the DBD-A and ssDNA nucleotide (non-specific short-ranged interaction) and the evolution time, τevolution, for the non-specific complex to evolve as the specific hRPA-ssDNA complex. (TIFF) [file pone.0278396.s003.tiff]

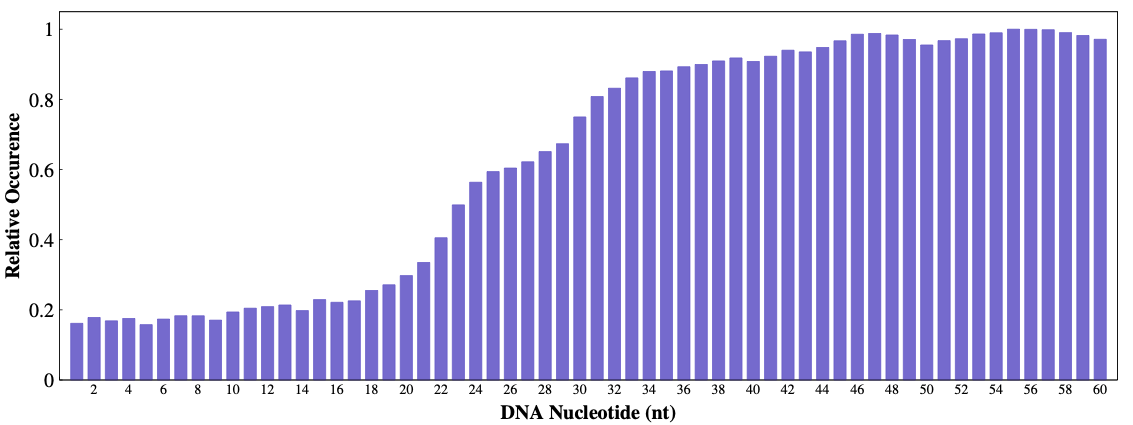

Supplement: S4 Fig — The relative occurrence of each nucleotide on hRPA surface in the ensemble of hRPA-ssDNA bound states as a function of DNA nucleotide. (TIFF) [file pone.0278396.s004.tiff]

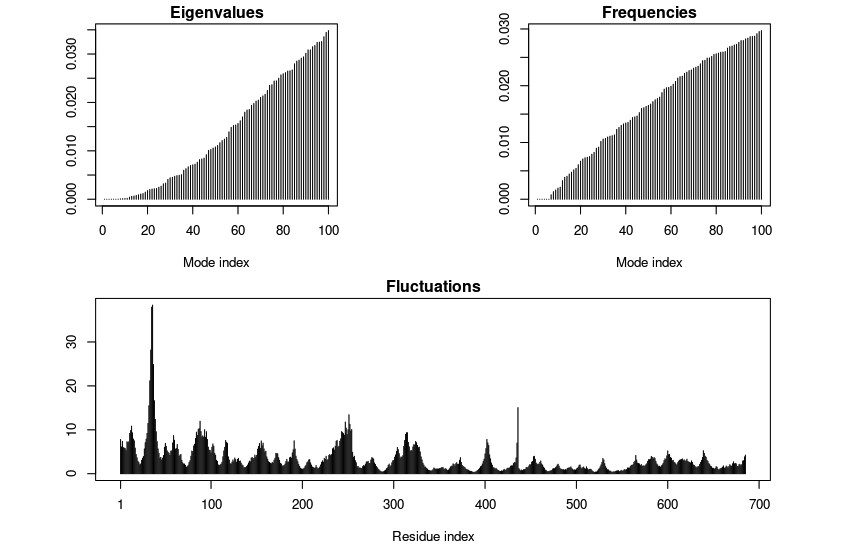

Supplement: S5 Fig — Eigenvalues, frequencies and fluctuations of normal modes for apo hRPA at ϵinter = 0.3 kcal/mol. Fluctuations are calculated based on averages for all modes weighted by eigen values. (TIFF) [file pone.0278396.s005.tiff]

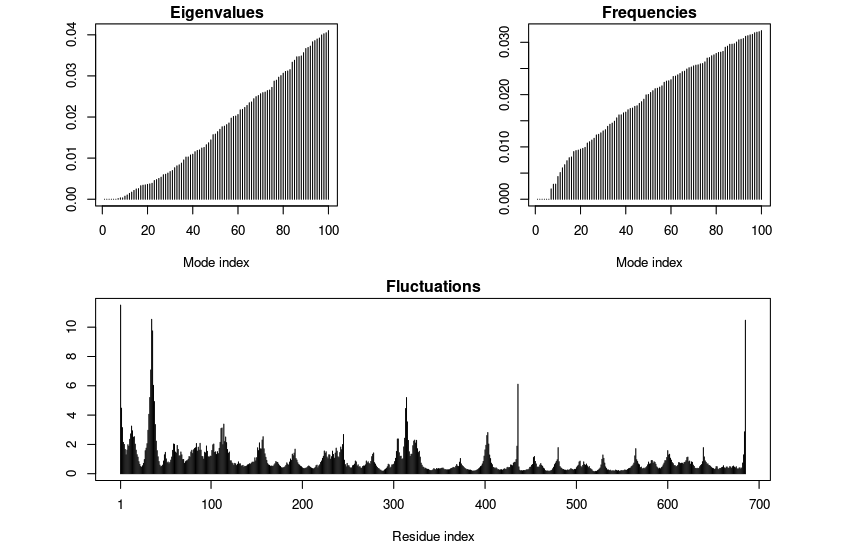

Supplement: S6 Fig — Eigenvalues, frequencies and fluctuations of normal modes for hRPA-ssDNA complex at ϵinter = 0.3 kcal/mol. Fluctuations are calculated based on averages for all modes weighted by eigen values. (TIFF) [file pone.0278396.s006.tiff]

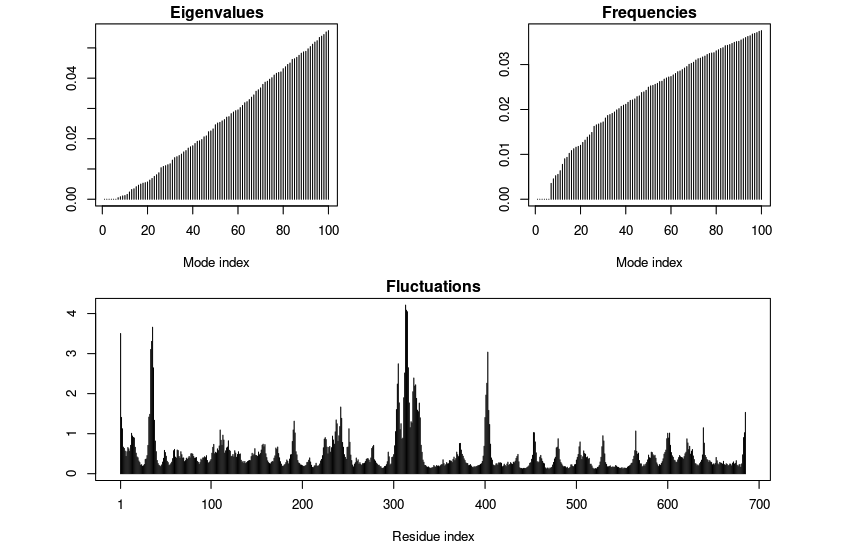

Supplement: S7 Fig — Eigenvalues, frequencies and fluctuations of normal modes for apo hRPA at ϵinter = 1.0 kcal/mol. Fluctuations are calculated based on averages for all modes weighted by eigen values. (TIFF) [file pone.0278396.s007.tiff]

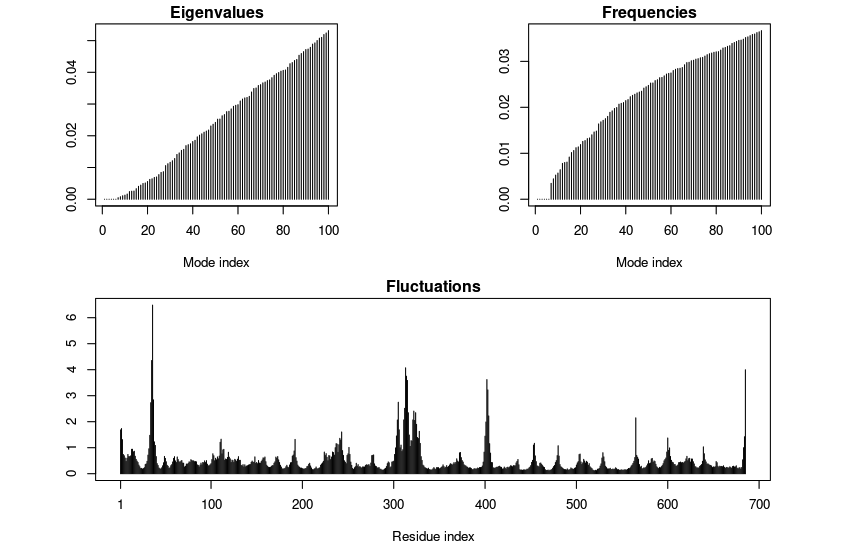

Supplement: S8 Fig — Eigenvalues, frequencies and fluctuations of normal modes for hRPA-ssDNA complex at ϵinter = 1.0 kcal/mol. Fluctuations are calculated based on averages for all modes weighted by eigen values. (TIFF) [file pone.0278396.s008.tiff]

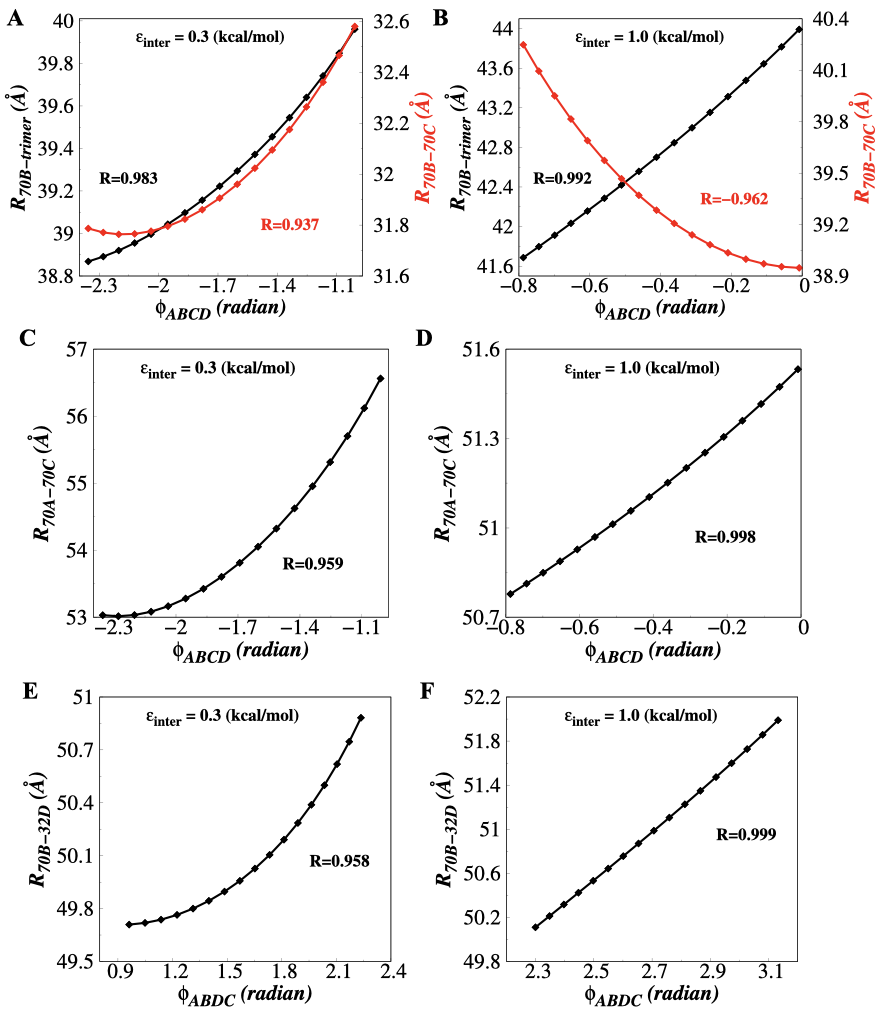

Supplement: S9 Fig — (A, B) The distances between DBD-B and trimer core, R70B-trimer (Å) (black color) and between DBD-B and DBD-C, R70B-70C (Å) (red color) as a function of the rotation around an imaginary axis connecting DBDs B and C (dihedral angle formed between the ABCD domains ΦABCD) for (A) flexible hRPA model and (B) rigid hRPA model. (C, D) The distances between DBD-A and DBD-C, R70A-70C (Å) (black color) as a function ΦABCD for (C) flexible hRPA model and (D) rigid hRPA model. (E, F) The distances between DBD-B and DBD-D, R70B-32D (Å) (black color) as a function ΦABDC for (E) flexible hRPA model and (F) rigid hRPA model. (TIFF) [file pone.0278396.s009.tiff]
