# Supplementary material for: Interdomain dynamics in human Replication Protein A regulates kinetics and thermodynamics of its binding to ssDNA
Source: PLoS One. 2023 Jan 19;18(1):e0278396. doi: 10.1371/journal.pone.0278396 (PMC9851514; doi:10.1371/journal.pone.0278396)
Supplement: S1 Table — Mass and radius of Amino Acid Residues. (DOCX) [file pone.0278396.s010.docx]

**S1 Table. The parameters used to model protein**

Mass and radius of Amino Acid Residues

| Amino acid | Mass (Da) | Radius of $C_{\alpha}$atom ($Å$) |
| --- | --- | --- |
| Glycine (G) | 75.067 | 2.0 |
| Alanine (A) | 89.094 | 2.0 |
| Valine (V) | 117.148 | 2.0 |
| Leucine (L) | 131.175 | 2.0 |
| Isoleucine (I) | 131.175 | 2.0 |
| Serine (S) | 105.093 | 2.0 |
| Threonine (T) | 119.119 | 2.0 |
| Cysteine (C) | 121.154 | 2.0 |
| Methionine (M) | 149.208 | 2.0 |
| Proline (P) | 115.132 | 2.0 |
| Asparagine (N) | 132.119 | 2.0 |
| Glutamine (Q) | 146.146 | 2.0 |
| Phenylalanine (F) | 165.192 | 2.0 |
| Tryptophan (W) | 204.228 | 2.0 |
| Histidine (H) | 155.156 | 2.0 |
| Tyrosine (Y) | 181.191 | 2.0 |
| Arginine (R) | 174.203 | 2.0 |
| Aspartic Acid (D) | 133.104 | 2.0 |
| Lysine (K) | 146.189 | 2.0 |
| Glutamic acid (E) | 147.131 | 2.0 |
