# Supplementary material for: Interdomain dynamics in human Replication Protein A regulates kinetics and thermodynamics of its binding to ssDNA
Source: PLoS One. 2023 Jan 19;18(1):e0278396. doi: 10.1371/journal.pone.0278396 (PMC9851514; doi:10.1371/journal.pone.0278396)
Supplement: S2 Table — (A) Mass and radius of nucleotide components. (B) Table of equilibrium bond lengths (r0), bend angles (θ0) and torsion angles (ϕ0) in the coarse-grained DNA model. The phosphate and sugar of the adjacent site in the 5’ direction are denoted by P(5’) and S(5’) respectively. Similarly, the phosphate and sugar in the 3’ direction are denoted by P(3’) and S(3’) respectively. (C) Values of strengths ϵij for base-stacking interaction in the 3SPN.2 DNA model. ↑ and ↓ denotes the sense and anti-sense strands respectively. 3’ ↑ and ↓ 5’ denotes adjacent bases in the 3’ and 5’ directions respectively. (D) The values of equilibrium distances (σij) and equilibrium angles (θBS0) for base stacking interactions. ↑ and ↓ denotes the sense and anti-sense strands respectively. 3’ ↑ and ↓ 5’ denotes adjacent bases in the 3’ and 5’ directions respectively. (DOCX) [file pone.0278396.s011.docx]

**S2 Table. The parameters used to model ssDNA**

1. Mass and radius of nucleotide components

| Nucleotide components | Mass (amu) | Radius ($Å$) |
| --- | --- | --- |
| Phosphate | 94.97 | 2.25 |
| Sugar | 83.11 | 3.20 |
| Adenine (A) | 134.1 | 2.70 |
| Thymine (T) | 125.1 | 3.55 |
| Guanine (G) | 150.1 | 2.45 |
| Cytosine (C) | 110.1 | 3.20 |

1. Table of equilibrium bond lengths${(r}^{0}$), bend angles ($\theta^{0}$) and torsion angles ($\phi^{0}$) in the coarse-grained DNA model [10]. The phosphate and sugar of the adjacent site in the 5$'$ direction are denoted by P(5$'$) and S(5$'$) respectively. Similarly, the phosphate and sugar in the 3$'$ direction are denoted by P(3$'$) and S(3$'$) respectively.

| Bond | $r^{0} (Å)$ | Bend | $\theta^{0}(^{\circ})$ | Torsion | $\phi^{0}(^{\circ})$ | $\sigma_{\phi}$ |
| --- | --- | --- | --- | --- | --- | --- |
| P(5$'$)$-$S | 3.899 | S$-$P$-$S | 94.49 | (5$'$)P$-$S$-$P$-$S(3$'$) | -154.79 | 0.30 |
| S$-$P(3$'$) | 3.559 | P$-$S$-$P | 120.15 | (5$'$)S$-$P$-$S$-$P(3$'$) | -179.17 | 0.30 |
| S$-$A | 4.670 | P$-$S$-$A | 103.53 |  |  |  |
| S$-$T | 4.189 | P$-$S$-$T | 92.06 |  |  |  |
| S$-$G | 4.829 | P$-$S$-$G | 107.40 |  |  |  |
| S$-$C | 3.844 | P$-$S$-$C | 103.79 |  |  |  |
|  |  | A$-$S$-$P | 112.07 |  |  |  |
|  |  | T$-$S$-$P | 116.68 |  |  |  |
|  |  | G$-$S$-$P | 110.12 |  |  |  |
|  |  | C$-$S$-$P | 110.33 |  |  |  |

1. Values of strengths $\epsilon_{ij}$ for base-stacking interaction in the 3SPN.2 DNA model [10]. $\uparrow$ and $\downarrow$ denotes the sense and anti-sense strands respectively. 3$'$ $\uparrow$ and $\downarrow$ 5$'$ denotes adjacent bases in the 3$'$ and 5$'$ directions respectively.

| Base 3$'$ $\uparrow$ $\epsilon_{ij}$ (kJ/mol) | | | | | |
| --- | --- | --- | --- | --- | --- |
|  |  | A | T | G | C |
|  | A | 14.39 | 14.34 | 13.25 | 14.51 |
| Base 5$'$ $\uparrow$ | T | 10.37 | 13.36 | 10.34 | 12.89 |
|  | G | 14.81 | 15.57 | 14.93 | 15.39 |
|  | C | 11.42 | 12.79 | 10.52 | 13.24 |

1. The values of equilibrium distances ($\sigma_{ij}$) and equilibrium angles ($\theta_{BS}^{0}$) for base stacking interactions [10]. $\uparrow$ and $\downarrow$ denotes the sense and anti-sense strands respectively. 3$'$ $\uparrow$ and $\downarrow$ 5$'$ denotes adjacent bases in the 3$'$ and 5$'$ directions respectively.

| Base 3$'$ $\uparrow$ | | | | | | | | | | |
| --- | --- | --- | --- | --- | --- | --- | --- | --- | --- | --- |
|  |  |  | $\sigma_{ij} (Å)$ |  |  |  |  | $\theta_{BS}^{0} (^{\circ})$ |  |  |
|  |  | A | T | G | C |  | A | T | G | C |
|  | A | 3.716 | 3.675 | 3.827 | 3.975 | A | 101.15 | 85.94 | 105.26 | 90.26 |
| Base 5$'$ $\uparrow$ | T | 4.238 | 3.984 | 4.416 | 4.468 | T | 101.59 | 89.50 | 104.31 | 90.82 |
|  | G | 3.576 | 3.598 | 3.664 | 3.822 | G | 100.89 | 84.83 | 105.48 | 90.18 |
|  | C | 3.859 | 3.586 | 4.030 | 3.957 | C | 115.95 | 101.51 | 119.32 | 104.49 |
