# Supplementary material for: Interdomain dynamics in human Replication Protein A regulates kinetics and thermodynamics of its binding to ssDNA
Source: PLoS One. 2023 Jan 19;18(1):e0278396. doi: 10.1371/journal.pone.0278396 (PMC9851514; doi:10.1371/journal.pone.0278396)
Supplement: S3 Table — (A) The experimental values of strength ϵijAA-B for specific -π−π stacking interactions between different aromatic residue—nucleobase pairs (as reported by Rutledge et al.). The table values are adopted from the previous studies on the binding of proteins with ssDNA (references in the supplementary text). (B) The values of strength ϵijHydrogen for hydrogen bonding interactions between non-aromatic Cα bead and nucleobase pairs. (DOCX) [file pone.0278396.s012.docx]

**S3 Table. The parameters used to model protein - ssDNA interactions**

1. The experimental values of strength $\epsilon_{ij}^{AA-B}$ for specific $\pi-\pi$ stacking interactions between different aromatic residue $-$ nucleobase pairs (as reported by Rutledge *et al.* [12]). The table values are adopted from the previous studies on the binding of proteins with ssDNA and protein with ssRNA (references in the supplementary text).

|  | A | T | G | C |
| --- | --- | --- | --- | --- |
| PHE | 3.3 | 2.8 | 3.0 | 1.7 |
| TYR | 3.3 | 2.3 | 2.9 | 1.4 |
| TRP | 3.1 | 4.3 | 3.9 | 2.4 |
| HIS | 2.2 | 2.5 | 2.2 | 1.4 |

1. The values of strength $\epsilon_{ij}^{Hydrogen}$ for hydrogen bonding interactions between non-aromatic $C_{\alpha}$ bead and nucleobase pairs.

|  | A | T | G | C |
| --- | --- | --- | --- | --- |
| GLY | 0.30 | 0.75 | 0.21 | 0.60 |
| ALA | 0.06 | 0.45 | 0.04 | 0.35 |
| VAL | 0.04 | 0.15 | 0.26 | 0.30 |
| LEU | 0.01 | 0.10 | 0.04 | 0.10 |
| ILE | 0.04 | 0.10 | 0.36 | 0.10 |
| SER | 0.30 | 1.50 | 0.61 | 1.00 |
| THR | 0.19 | 0.50 | 0.51 | 0.60 |
| CYS | 0.01 | 0.01 | 0.01 | 0.01 |
| MET | 0.07 | 0.41 | 0.06 | 0.15 |
| PRO | 0.01 | 0.15 | 0.01 | 0.01 |
| ASN | 0.02 | 0.41 | 0.18 | 0.20 |
| GLN | 0.01 | 0.41 | 0.04 | 0.41 |
