# Supplementary material for: Interdomain dynamics in human Replication Protein A regulates kinetics and thermodynamics of its binding to ssDNA
Source: PLoS One. 2023 Jan 19;18(1):e0278396. doi: 10.1371/journal.pone.0278396 (PMC9851514; doi:10.1371/journal.pone.0278396)
Supplement: S4 Table — The model has previously been used to capture the bound state conformations of a number of protein-ssDNA complexes (including SSB-ssDNA complex) irrespective of the length and sequences of ssDNA. A list of the PDB IDs, ssDNA sequence, Number of nucleotides and the RMSD value predicted from our model with respect to the relevant crystal structures is given. (DOCX) [file pone.0278396.s013.docx]

**S4 Table**. **Protein-ssDNA model Validation**

The model has previously been used to capture the bound state conformations of a number of protein-ssDNA complexes (including SSB-ssDNA complex) irrespective of the length and sequences of ssDNA. A list of the PDB IDs, ssDNA sequence, Number of nucleotides and the RMSD value predicted from our model with respect to the relevant crystal structures is given.

| PDB ID | ssDNA sequence | #Nucleotides | RMSD ($Å$) |
| --- | --- | --- | --- |
| 4GNX | TTTTTTTTTTTTTTTTTTTTTTTTT | 25 | 0.849 |
| 6I52 | TTTTTTTTTTTTTTTTTTTT | 20 | 0.940 |
| 2LTT | TTTTTTTTTTTTTTTTT | 17 | 0.885 |
| 2CCZ | TTTTTTTTTTTTTTT | 15 | 0.699 |
| 4OU6 | TTTTTTTTTT | 10 | 2.103 |
| 5ODL | TTTTTTTTT | 9 | 1.011 |
| 2ES2 | TTTTTT | 6 | 0.550 |
| 2MNA | TTTTTT | 6 | 0.623 |
| 2C62 | TTTTTTTTTTTTTTTTTTTG | 20 | 0.722 |
| 5ZG9 | TTTTTTTTTTTTTTTTTTTG | 20 | 0.676 |
| 1EYG | Poly C | 35 | 0.972 |
| 1JMC | CCCCCCCC | 8 | 0.852 |
| 1S40 | GTGTGGGTGTG | 11 | 0.759 |
| 2UP1 | TAGGGTTAGGG | 11 | 0.930 |
| 3VKE | ACCCCA | 6 | 0.879 |
| 1QZH | GGTTAC | 6 | 0.637 |
| 1ZZI | CTCCCC | 6 | 0.802 |
| 2KN7 | CAGTGGCTGA | 10 | 0.572 |
| 2MAP | TGTCAAA | 7 | 0.979 |
| 4HIO | GGTAACGGT | 9 | 0.690 |
| 4HJA | ACGGTTACGGT | 11 | 0.667 |
| 5ORQ | TTAGGGTTAG | 10 | 0.502 |
| 5USB | GGTTACGGT | 9 | 0.636 |
| 6BUX | AATCCCAAA | 9 | 0.713 |
| 6KBS | CGGTCGATTC | 10 | 0.799 |
