# Supplementary material for: Interdomain dynamics in human Replication Protein A regulates kinetics and thermodynamics of its binding to ssDNA
Source: PLoS One. 2023 Jan 19;18(1):e0278396. doi: 10.1371/journal.pone.0278396 (PMC9851514; doi:10.1371/journal.pone.0278396)
Supplement: S5 Table — (DOCX) [file pone.0278396.s014.docx]

**S5 Table. The list of citations from where regarding the model parameters used for modeling of protein, ssDNA and protein-ssDNA interactions in this study.**

| **Model Details and Parameters** | **Sources (In supplementary references)** |
| --- | --- |
| Protein Model (Go Like Model) | [1,2] |
| ssDNA Model (3SPN.2 Model) | [10] |
| Protein-ssDNA interaction | [9,12,15] |
