# Supplementary material for: Interdomain dynamics in human Replication Protein A regulates kinetics and thermodynamics of its binding to ssDNA
Source: PLoS One. 2023 Jan 19;18(1):e0278396. doi: 10.1371/journal.pone.0278396 (PMC9851514; doi:10.1371/journal.pone.0278396)
Supplement: S1 File — (DOCX) [file pone.0278396.s019.docx]

**Supporting Information References**

**References for Supporting Information citations.**

1. Bhattacherjee A, Krepel D, Levy Y. Coarse-grained models for studying protein diffusion along DNA. WIREs Comput Mol Sci. 2016;6: 515–531. doi:https://doi.org/10.1002/wcms.1262

2. Clementi C, Nymeyer H, Onuchic JN. Topological and energetic factors: what determines the structural details of the transition state ensemble and “en-route” intermediates for protein folding? An investigation for small globular proteins. J Mol Biol. 2000;298: 937–953.

3. Bhattacherjee A, Levy Y. Search by proteins for their DNA target site: 1. The effect of DNA conformation on protein sliding. Nucleic Acids Res. 2014/10/16. 2014;42: 12404–12414.

4. Bhattacherjee A, Levy Y. Search by proteins for their DNA target site: 2. The effect of DNA conformation on the dynamics of multidomain proteins. Nucleic Acids Res. 2014;42: 12415–12424.

5. Dey P, Bhattacherjee A. Role of Macromolecular Crowding on the Intracellular Diffusion of DNA Binding Proteins. Sci Rep. 2018;8: 844.

6. Dey P, Bhattacherjee A. Mechanism of Facilitated Diffusion of DNA Repair Proteins in Crowded Environment: Case Study with Human Uracil DNA Glycosylase. J Phys Chem B. 2019;123: 10354–10364.

7. Dey P, Bhattacherjee A. Disparity in anomalous diffusion of proteins searching for their target DNA sites in a crowded medium is controlled by the size, shape and mobility of macromolecular crowders. Soft Matter. 2019;15: 1960–1969.

8. Dey P, Bhattacherjee A. Structural Basis of Enhanced Facilitated Diffusion of DNA-Binding Protein in Crowded Cellular Milieu. Biophys J. 2020;118: 505–517.

9. Mondal A, Bhattacherjee A. Mechanism of Dynamic Binding of Replication Protein A to ssDNA. J Chem Inf Model. 2020;60: 5057–5069.

10. Hinckley DM, Freeman GS, Whitmer JK, de Pablo JJ. An experimentally-informed coarse-grained 3-Site-Per-Nucleotide model of DNA: structure, thermodynamics, and dynamics of hybridization. J Chem Phys. 2013;139: 144903.

11. Lequieu J, Córdoba A, Schwartz DC, de Pablo JJ. Tension-Dependent Free Energies of Nucleosome Unwrapping. ACS Cent Sci. 2016;2: 660–666.

12. Rutledge LR, Campbell-Verduyn LS, Hunter KC, Wetmore SD. Characterization of nucleobase-amino acid stacking interactions utilized by a DNA repair enzyme. J Phys Chem B. 2006;110: 19652–19663.

13. Wilson KA, Kellie JL, Wetmore SD. DNA-protein π-interactions in nature: abundance, structure, composition and strength of contacts between aromatic amino acids and DNA nucleobases or deoxyribose sugar. Nucleic Acids Res. 2014;42: 6726–6741.

14. Mishra G, Levy Y. Molecular determinants of the interactions between proteins and ssDNA. Proc Natl Acad Sci. 2015;112: 5033–5038.

15. Pal A, Levy Y. Structure, stability and specificity of the binding of ssDNA and ssRNA with proteins. PLOS Comput Biol. 2019;15: e1006768.

16. Chu X, Wang J. Position-, disorder-, and salt-dependent diffusion in binding-coupled-folding of intrinsically disordered proteins. Phys Chem Chem Phys. 2019;21: 5634–5645.

17. Kumar S, Rosenberg JM, Bouzida D, Swendsen RH, Kollman PA. THE weighted histogram analysis method for free-energy calculations on biomolecules. I. The method. J Comput Chem. 1992;13: 1011–1021.

18. Veitshans T, Klimov D, Thirumalai D. Protein folding kinetics: timescales, pathways and energy landscapes in terms of sequence-dependent properties. Fold Des. 1997;2: 1–22.
